# A novel rare variants association test for binary traits in family-based designs via copulas

**DOI:** 10.1177/09622802231197977

**Published:** 2023-10-13

**Authors:** Houssou R. G. Dossa, Alexandre Bureau, Michel Maziade, Lajmi Lakhal-Chaieb, Karim Oualkacha

**Affiliations:** 1 Département de Mathématiques, Université du Québec à Montréal (UQAM) et, Québec, Canada; 2 Département de Médecine Sociale et Préventive, 4440 Université Laval, Québec, Canada; 3 Centre de Recherche CERVO, Quebec, Canada; 4 Département de Psychiatrie et Neuroscience, 4440 Université Laval, Québec, Canada; 5 Département de Mathématiques et Statistique, 4440 Université Laval, Québec, Canada

**Keywords:** Score test, rare variants, association tests, region-based tests, copulas

## Abstract

With the cost-effectiveness technology in whole-genome sequencing, more sophisticated statistical methods for testing genetic association with both rare and common variants are being investigated to identify the genetic variation between individuals. Several methods which group variants, also called gene-based approaches, are developed. For instance, advanced extensions of the sequence kernel association test, which is a widely used variant-set test, have been proposed for unrelated samples and extended for family data. Family data have been shown to be powerful when analyzing rare variants. However, most of such methods capture familial relatedness using a random effect component within the generalized linear mixed model framework. Therefore, there is a need to develop unified and flexible methods to study the association between a set of genetic variants and a trait, especially for a binary outcome. Copulas are multivariate distribution functions with uniform margins on the [0,1] interval and they provide suitable models to capture familial dependence structure. In this work, we propose a flexible family-based association test for both rare and common variants in the presence of binary traits. The method, termed novel rare variant association test (NRVAT), uses a marginal logistic model and a Gaussian Copula. The latter is employed to model the dependence between relatives. An analytic score-type test is derived. Through simulations, we show that our method can achieve greater power than existing approaches. The proposed model is applied to investigate the association between schizophrenia and bipolar disorder in a family-based cohort consisting of 17 extended families from Eastern Quebec.

## 1. Introduction

The identification of the association between single nucleotide variants (SNVs) with complex diseases is the main goal of genetic studies.^1^ In a genome-wide association studies (GWAS) framework, researchers generally focus on common causal variants with a minor allele frequency (MAF) ≥5% . Now, because of the cost-effectiveness of next-generation sequencing (NGS) technologies, hundreds of millions of genetic variants with mostly low MAF have been identified. Rare variants can be defined as genetic mutations that occur at low frequency in a population. They are variants with MAF <5%. Single variant tests that have been proposed in GWAS have generally low power when using rare genetic variants in NGS studies. To avoid the power loss issue, several methods such as the burden test,^2,3^ sequence kernel association test (SKAT),^4^ and their various combinations have been proposed.^6,5^ Sequence kernel machine-based association methods test for association between a set of rare/common variants and quantitative or binary phenotypes, and most of them are based on generalized linear mixed models.

In this study, we focus on family-based designs. Sampling relatives in sequencing studies may sometimes be more advantageous than sampling unrelated subjects.^7^ It has the advantage of enriching data sets for familial rare disease variants because of the segregation of alleles within pedigrees, and it may protect data analysis against population stratification issues. Moreover, sequencing data are subject to read (or sequence) errors, and the Mendelian pedigree information available when related individuals are sequenced can help to identify technological artefacts in the data and improve the protection against sequencing errors.^9,8,10^ For such reasons, several whole-genome sequencing (WGS) projects have been conducted for family-based cohorts to identify rare variants’ effects. For example, the National Heart, Lung, and Blood Institute’s Trans-Omics for Precision Medicine (TOPMed) program and the National Human Genome Research Institute’s Genome Sequencing Project, have performed WGS from more than 120,000 samples not only for population-based cohort but also for family studies.^5^ The Type 2 Diabetes (T2D-GENES) Consortium has been conducted in WGS from 20 large Mexican-American families containing multiple cases of Type 2 diabetes, which aims to detect rare coding variants associated with that disease.^11,12^

Several region-based sequence kernel association methods have been developed in the literature to analyse familial data. The methods can be broadly divided into two categories to handle the cluster dependencies induced by related subjects: The first category relies on marginal models linking the outcome to genetic predictors, and use the working-correlation trick to control for the within-family dependence structure through generalized estimating equations (GEE).^13,14^ GEE methods do not need to impose assumptions on the trait joint distribution within each family. Thus they are more robust to distribution misspecification and might be suitable for large-scale studies with complicated data design. However, the test from GEE can suffer from low power if the working covariance is not well calibrated, that is, if the family size is not uniform.^14^ The methods of the second category rely on conditional models, which capture the clustered structure by adding a random effect in the generalized linear mixed model (GLMM) framework.^17,15,16,6^ Most existing methods in this category are designed for continuous outcomes. To face this situation, a computationally efficient logistic linear mixed model association test (GMMAT) has been proposed by Chen et al.^18^ As GMMAT was originally developed for single-variant analysis, the authors, later extended the method to variant-set mixed model association tests (SMMAT) for binary traits.^5^

Other testing approaches that are computationally feasible regarding the family-based designs for binary traits in a region-based framework have been proposed by Saad and Wijsman.^19^ The method is termed allele frequency comparison (AFC) tests. Note that these tests were proposed firstly for single-variant analysis by Bourgain et al.^20^ and Choi et al.^21^ AFC tests are based on the difference in single nucleotide polymorphism (SNP) allele frequencies in the cases (affected) and controls (unaffected). They use multivariate random-effects with a covariance matrix implying the kinship matrix to account for family relationships between individuals. Unlike the linear mixed models, these tests do not need the kinship matrix to be positive semidefinite, which makes them interesting as they can allow the use of an estimated genetic relationship matrix (GRM) from genotype data, which might not be positive semidefinite. Although the AFC tests are useful, they cannot adjust for covariates. Moreover, they may suffer from type 1 error rate inflation when the correlation between SNPs is not estimated accurately (i.e. correlation computed using small sample sizes).

In this paper, we propose a flexible copula-based variant-set association test for binary phenotypes in family-based designs. Copulas are suitable models for modelling joint distributions since they allow for separation of the dependence and marginal distributions.^22–24^ That is, the proposed model, termed novel rare variant association test (NRVAT), uses (marginal) generalized link functions to relate the binary phenotype to both the covariates and genetic variants and models the dependence between relatives through a Gaussian copula with correlation matrix corresponding to the familial kinship matrix to describe the polygenic relationship between subjects of a same family. The proposed joint modelling approach has several advantages over current GLMM-based methods, such as GMMAT and SMMAT. For instance, the dependence structure can be captured using a different copula model (e.g. student t or chi-square copula), which allows for more flexible models. Because the association between the binary phenotype and both the covariates and genotypes can be captured using usual (marginal) Generalized linear models (GLM), this allows for simple interpretability of the phenotype–genotype association parameters. Moreover, the trait polygenic heritability is ‘margin-free’, in the sense that it is characterized by the copula alone.

The remainder of this paper is structured as follows. In Section 2, we formally describe the proposed statistical framework. Numerical studies are conducted to compare different models in Section 3. In Section 4, we apply the proposed method to real data. We discuss and conclude this paper in Section 5.

## 2. Data and model

Consider I families and for i=1,…,I , let $n_{i}$ be the size of the ith family. The total sample size is $N=\sum_{i=1}^{I}n_{i}$ . Let $Y_{ij}\in \{0,1\}$ be the binary phenotype under investigation for individual j in family i , for i=1,…,I and $j=1,\ldots ,n_{i}$ . We begin by specifying the (conditional) marginal distribution of $Y_{ij}$ , denoted $F(Y_{ij}|X_{ij},G_{ij})$ , where $X_{ij}=(1,X_{ij1},\ldots ,X_{ijs}{)}^{⊤}$ is a (s+1)×1 vector of covariates including the intercept and $G_{ij}=(G_{ij1},\ldots ,G_{ijr}{)}^{⊤}$ is a r×1 vector of genotypes coded as ( 0,1,2 ) from biallelic variants. Since the response variable is dichotomous, $F(Y_{ij}|X_{ij},G_{ij})$ is completely specified by $\mu_{ij}=P(Y_{ij}=1|X_{ij},G_{ij})$ . Thus, we relate the binary phenotype $Y_{ij}$ to $X_{ij}$ and $G_{ij}$ through a logistic regression model

(1) $$\text{logit}(\mu_{ij})=X_{ij}^{⊤}\gamma +G_{ij}^{⊤}\beta ,\;j=1,\ldots ,n_{i},\;i=1,\ldots ,I$$

where $\text{logit}(u)=\log\;[\frac{u}{1-u}],$ and $\gamma =(\gamma_{0},\gamma_{1},\ldots ,\gamma_{s}{)}^{⊤}$ and $\beta =(\beta_{1},\ldots ,\beta_{r}{)}^{⊤}$ are sets of regression coefficients. In a matrix notation, one has

$$\text{logit}(\mu_{i})=X_{i}\gamma +G_{i}\beta$$

where $\mu_{i}=(\mu_{i1},\ldots ,\mu_{in_{i}}{)}^{⊤}$ , $X_{i}$ is a $n_{i}\times (s+1)$ matrix with the jth row equal to $X_{ij}$ and $G_{i}$ is a $n_{i}\times r$ matrix with the jth row equal to $G_{ij}$ . The logit was taken element-wise of the entries of $\mu_{i}.$

We then specify a joint distribution for $Y_{i}=(Y_{i1},\ldots ,Y_{in_{i}}{)}^{⊤}$ based on a latent-variable model, which assumes a $n_{i}\times 1$ latent vector $Z_{i}=(Z_{i1},\ldots ,Z_{in_{i}}{)}^{⊤}$ such that

(2) $$Y_{ij}=\left\{ \begin{array}{ll} 1 & \text{if}\;Z_{i\;j}\leq \Phi^{-1}(\mu_{ij})\\ 0 & \text{otherwise} \end{array}\right.$$

with $Z_{i}∼N(0_{n_{i}},\Gamma_{i})$ , $\Gamma_{i}=h^{2}\Psi_{i}+(1-h^{2})I_{n_{i}}$ , $h^{2}$ measures the polygenic heritability, $\Psi_{i}$ is a matrix with entries reflecting the proportion of the genome that is shared identically by descent between subjects, and $I_{n_{i}}$ is the identity matrix of size $n_{i}$ . This leads to

$$Pr(Y_{i\;1}=1,\ldots ,Y_{i\;n_{i}}=1|X_{i},G_{i})=Pr(Z_{i\;1}\leq \Phi^{-1}(\mu_{i1}),\ldots ,Z_{i\;n_{i}}\leq \Phi^{-1}(\mu_{i\;n_{i}}))=C_{\Gamma_{i}}(\mu_{i\;1},\ldots ,\mu_{i\;n_{i}})$$

where

(3) $$C_{\Gamma }(u_{1},\ldots ,u_{d})=\Phi_{d}(\Phi^{-1}(u_{1}),\ldots ,\Phi^{-1}(u_{d})|\Gamma )$$

is a Gaussian copula, with $u_{j}\in [0,1],j=1,\ldots ,d$ , and $\Phi_{d}(.|\Gamma )$ is the d -dimensional Gaussian cumulative distribution function with mean zero and d×d correlation matrix Γ .

The role of the copula $C_{\Gamma_{i}}$ is to account for possible dependence between the residuals of the marginal models. In other words, the $n_{i}\times n_{i}$ variance–covariance matrix of the vector of residuals, $\Sigma_{i}:=Var(Y_{i}-\mu_{i})$ , is given as follows:

$$\Sigma_{i}^{jj}=\mu_{ij}(1-\mu_{ij}),\;\text{with}\;\Sigma_{i}^{jk}=C_{{\tilde{\Gamma }}_{i}^{jk}}(\mu_{ij},\mu_{ik})-\mu_{ij}\mu_{ik}$$

where ${\tilde{\Gamma }}_{i}^{jk}$ is a 2×2 correlation matrix with the out-of-diagonal element equals to the (j,k) element of $\Gamma_{i}$ , $\Gamma_{i}^{jk}$ .

Motivated by such advantages provided by the proposed joint modelling approach, we suggest building a score-type test statistic for phenotype–genotype association based on the marginal logistic regression model and adjusting for possible dependence between marginal residuals via the copula model. The proposed score-type test procedure is detailed in the next section.

### 2.1. Inference procedure

Under the proposed model, the association between the r variants and the phenotype can be tested by evaluating the null hypothesis $H_{0}:\beta =0_{r}$ .

Deriving the test statistic from the complete log-likelihood function induces complex formulae that prevent the practical implementation of the test. In this work, we consider an alternative approach and propose to derive the test statistic under the independent working assumption.^26,25^ The log-likelihood function is then written as

(4) $$l_{ind}(\beta ,\gamma )=\sum_{i=1}^{I}\sum_{j=1}^{n_{i}}y_{ij}(X_{ij}^{⊤}\gamma +\;G_{ij}^{⊤}\beta )-\log\;\{1+e^{X_{ij}^{⊤}\gamma +\;G_{ij}^{⊤}\beta }\}$$

We adopt the variance-component (VC) hypothesis testing technique, which is a standard approach in the region-based association framework. That is, we treat β as a random vector following an arbitrary distribution H with mean zero and variance–covariance matrix τW , where τ is a VC scalar and $W=diag(w_{1},\ldots ,w_{r})$ is a r×r diagonal matrix of a priori weights to be used for the r variants, here we used MAF-based weights. Thus, testing for $H_{0}:\beta =0_{r}$ is equivalent to testing for τ=0 .

Under such a framework, one has

$$l_{VC}(\tau ,\gamma )=\log\;\left\{\int e^{l_{ind}(\beta ,\gamma )}dH(\beta |\tau )\right\}$$

Following the same rationale as in the derivation of the SKAT[^15^ and VC Copula-based score statistics of Lakhal-Chaieb et al.^7^ (refer to Supplemental materials for details), we show that

(5) $$\frac{\partial l_{\mathrm{VC}}(\tau ,\gamma )}{\partial \tau }{|}_{\tau =0}=\frac{1}{2}\left[(Y-\mu {)}^{⊤}K(Y-\mu )-\text{tr}(K\Delta )\right]$$

where $Y=(Y_{1}^{⊤},\ldots ,Y_{I}^{⊤}{)}^{⊤}$ , $\mu =(\mu_{1}^{⊤},\ldots ,\mu_{I}^{⊤}{)}^{⊤}$ , and $\Delta =\text{diag}(\Delta_{1},\ldots ,\Delta_{I})$ and $\Delta_{i}=\text{diag}[\mu_{i}(1-\mu_{i})]$ , i=1,…,I . Here, K is a N×N Kernel matrix that can be written as

(6) $$K=\left(\begin{array}{cccc} K_{11} & K_{12} & \cdots & K_{1I}\\ \vdots & \vdots & \cdots & \vdots \\ K_{I1} & K_{I2} & \cdots & K_{II} \end{array}\right)$$

where $K_{ii^{\prime }}$ is a $n_{i}\times n_{i^{\prime }}$ sub-matrix with the $\left(j,j^{\prime }\right)$ entry equal to $\sum_{l=1}^{r}w_{l}G_{ijl}G_{i^{\prime }j^{\prime }l}$ .

The variability of the second term of the right-hand side of (5) is negligible compared with the first one. This prompts us to consider the test statistic $Q=(Y-\hat{\mu }{)}^{⊤}K(Y-\hat{\mu })$ , where $\hat{\mu }$ is the estimator of μ under the null hypothesis. Explicitly, one has $\hat{\mu }=({\hat{\mu }}_{1}^{⊤},\ldots ,{\hat{\mu }}_{I}^{⊤}{)}^{⊤}$ , where ${\hat{\mu }}_{i}=g_{i}(\hat{\gamma }),g_{i}(\gamma )={\text{logit}}^{-1}(X_{i}\gamma )$ , i=1,…,I and $\hat{\gamma }={\text{argmax}}_{\gamma }\;l_{\text{ind}}(0,\gamma )$ . In Appendix A, we show that

$$\sqrt{I}(\hat{\gamma }-\gamma )=A^{-1}\frac{1}{\sqrt{I}}\sum_{i=1}^{I}S_{i}(\gamma )+o_{p}(1)$$

where $A={\text{lim}}_{I\to \infty }I^{-1}\sum_{i=1}^{I}X_{i}^{⊤}\Delta_{i}X_{i}$ and $S_{i}(\gamma )=X_{i}^{⊤}[Y_{i}-g_{i}(\gamma )]$ . Therefore, one has $\sqrt{I}(\hat{\gamma }-\gamma )∼N(0,A^{-1}BA^{-1})$ , where $B={\text{lim}}_{I\to \infty }I^{-1}\sum_{i=1}^{I}X_{i}^{⊤}\Sigma_{i}X_{i}$ can be consistently estimated by $I^{-1}\sum_{i=1}^{I}S_{i}(\gamma )S_{i}(\gamma {)}^{⊤}$ .

In Appendix B, we show that the variance–covariance matrix of $Y_{i}-{\hat{\mu }}_{i}$ is

$$\Omega_{ii}=E[(Y_{i}-{\hat{\mu }}_{i})(Y_{i}-{\hat{\mu }}_{i}{)}^{⊤}]=\Sigma_{i}-\frac{1}{I}\Delta_{i}A^{-1}X_{i}^{⊤}\Sigma_{i}-\frac{1}{I}\Sigma_{i}X_{i}A^{-1}X_{i}^{⊤}\Delta_{i}+\frac{1}{I}\Delta_{i}X_{i}A^{-1}BA^{-1}\Delta_{i}X_{i}$$

and that covariance matrix of $Y_{i}-{\hat{\mu }}_{i}$ and $Y_{i^{\prime }}-{\hat{\mu }}_{i^{\prime }}$ is

$$\Omega_{ii^{\prime }}=E[(Y_{i}-{\hat{\mu }}_{i})(Y_{i^{\prime }}-{\hat{\mu }}_{i^{\prime }}{)}^{⊤}]=-\frac{1}{I}\Delta_{i}X_{i}A^{-1}X_{i}^{⊤}\Sigma_{i}-\frac{1}{I}\Sigma_{i^{\prime }}X_{i^{\prime }}A^{-1}X_{i}^{⊤}\Delta_{i}+\frac{1}{I}\Delta_{i}X_{i}A^{-1}BA^{-1}X_{i^{\prime }}^{⊤}\Delta_{i^{\prime }}$$

Therefore, the variance–covariance matrix of $Y-\hat{\mu }$ is

$$\Omega =\left(\begin{array}{cccc} \Omega_{11} & \Omega_{12} & \cdots & \Omega_{1I}\\ \vdots & \vdots & \cdots & \vdots \\ \Omega_{I1} & \Omega_{I2} & \cdots & \Omega_{II} \end{array}\right)$$

Hence, the distribution of the test statistic Q under the null hypothesis can be approximated by a weighted mixture of chi-squared distributions $\sum_{n=1}^{N}\theta_{n}\chi_{1,n}^{2}$ , where $\left(\theta_{1},\ldots ,\theta_{r}\right)$ are the eigenvalues of $\Omega^{1/2}K\Omega^{1/2}$ and $\chi_{1,n}^{2}$ are independent $\chi_{1}^{2}$ random variables. In practice, Ω involves $h^{2}$ , which is unknown. In this work, we replace $h^{2}$ by its estimator obtained by maximizing, computed under the null hypothesis $H_{0}:\beta =0_{r},$ the pairwise log-likelihood which formulae is given by

$$\sum_{i=1}^{I}\sum_{1\leq j\neq j^{\prime }\leq n_{i}}\log\;[P(Y_{ij}=y_{ij},Y_{ij^{\prime }}=y_{ij^{\prime }}|X_{ij},X_{ij^{\prime }})]$$

See Appendix A for more details.

### 2.2. Generalizations

In this work, the dependence between the outcomes $\left(Y_{i1},\ldots ,Y_{in_{i}}\right)$ is modelled via a Gaussian copula. The latter is used to express the variance–covariance matrix $\Sigma_{i}$ and to estimate the polygenic heritability parameter $h^{2}$ . Therefore, one may generalize the derived test to any elliptic copula (^23^, chapter 4) without any loss of generality. The robustness of the derived test to the specification of the underlying copula is well-investigated in the simulation studies section. The Kernel matrix K in equation (6) is known in the literature as the linear kernel matrix.^4^ However, several other choices such as the quadratic and Gaussian kernels can be incorporated in the derived test, without loss of generality.^27^ In the simulations, their impact is investigated on both the type I error and the power of the proposed test.

## 3. Simulations

Simulations were carried out to validate and compare the performance of the proposed method, NRVAT, with three existing set-based rare-variant association methods dealing with binary phenotype in presence of families, namely SMMAT^5^; gSKAT^14^ and AFC tests.^19^ As mentioned previously, SMMAT is a GLMM method which captures family relationships via a random effect; gSKAT is a GEE-based association test which controls for familial relationships via the working-correlation trick; AFC test is a score-type association test based on the difference of SNP allele frequencies in the cases (affected) versus controls (unaffected) and uses a multivariate random effect to account for family relationships between individuals. The methods comparison is based on empirical type I error rate and power.

### 3.1. Data generation

This section outlines the detailed steps pursued to generate simulated data for the methods’ evaluation.

*Genotypes*: we used SIMULATE3 computer program,^28^ which allows simulation of genotypes of a set of linked SNPs in family members. In all simulations, we set the number of families to be I=120 , with 40 families of two parents and one child, 40 families of two parents and two children, and 40 families of three generations where there are eight subjects per family (i.e. 2 cousins with their 4 parents and 2 grandparents). This leads to a total of N=600 individuals (240 males and 360 females; 320 founders and 280 nonfounders) per each generated data. We set the number of simulated SNPs for each subject to be r=20 , with MAFs ranging between 0.003 and 0.01 . We assumed linkage disequilibrium between two adjacent SNPs to be $d^{2}=0.16$ (i.e. $d^{2}$ is the squared correlation coefficient between two adjacent SNPs).

*Covariates*: in all simulations, we considered two covariates associated with the binary response: a continuous covariate, $X_{1}∼Uniform[0,1]$ , and a binary covariate, $X_{2}∼Bernoulli(0.2)$ . The marginal effects of $X_{1}$ and $X_{2}$ on the outcome were set to $\gamma_{1}=\gamma_{2}=1$ , respectively. The intercept coefficient was set to $\gamma_{0}=-2$ .

*Binary phenotype*: we considered three settings to generate Y in order to conduct type I error and power comparisons with the competing methods. In Setting 1, Y was generated based on our copula model (2). In Setting 2, Y was simulated based on the GLMM model of the SMMAT approach. Finally, Setting 3 aimed to assess the robustness of the proposed copula approach with respect to miss-specification of the true copula describing the dependence structure between subjects of the same family. The three settings are described in detail as follows:

*Setting 1 (data generation from our model):* For each family i∈{1,…,I} , we simulated the response variable of the $n_{i}$ subjects following our copula model (2). The data generation steps are given as follows:

1. generate $n_{i}\times r$ matrix of genotypes $G_{i}$ from Simulate3;

2. set the first column of $n_{i}\times 3$ matrix $X_{i}$ to be the vector of ones, then generate entries of its additional two columns from the uniform distribution over [0,1] and a Bernoulli distribution with probability of success equals to 0.2 , respectively;

3. calculate the $n_{i}\times 1$ vector $\text{logit}(\mu_{i})=X_{i}\gamma +G_{i}\beta$ ;

4. generate $Z_{i}∼N(0_{n_{i}},\Gamma_{i}),$ with $\Gamma_{i}=h^{2}\Psi_{i}+(1-h^{2})I_{n_{i}}$ ;

5. generate $Y_{ij}=\left\{ \begin{array}{ll} 1 & \text{if}\;Z_{ij}\leq \Phi^{-1}(\mu_{ij})\\ 0 & \text{otherwise},\;\text{for}\;j=1,\ldots ,n_{i} \end{array}\right.$

*Setting 2 (data generation from a GLMM model):* We generated the response variable based on the GLMM model for family i∈{1,…,I} , as follows:

$$\text{logit}(\mu_{i})=X_{i}\gamma +G_{i}\beta +b_{i}$$

where $b_{i}∼N(0_{n_{i}},h^{2}\Psi_{i})$ , $G_{i}$ and $X_{i}$ are defined as in Setting 1. $\Psi_{i}$ is twice the ith family theoretical kinship matrix, and $h^{2}$ is the polygenic trait heritability.

*Setting 3 (copula miss-specification):* To investigate the robustness of the derived test to the miss-specification of the underlying/true copula, we conducted two scenarios in which the simulated data were generated from either the student t or chi-square copula models. However, our proposed Gaussian copula model given in (3) was fitted to the simulated data in order to derive p -values. More precisely, in both scenarios, for family i , the response variable was generated following the same steps of Setting 1, except Step 4 where $Z_{i}$ was generated following a multivariate student t with 3 degrees of freedom (df) and correlation matrix $\Gamma_{i}$ , in Scenario 1. In Scenario 2, $Z_{i}$ was simulated from a chi-square copula distribution, as follows: we first generated ${\tilde{Z}}_{i}∼N(0_{n_{i}},\Gamma_{i})$ , then we set $U_{ij}=\text{sign}\;({\tilde{Z}}_{ij}+a)\;\{\Phi \;({\tilde{Z}}_{ij}+2a)+\Phi \;({\tilde{Z}}_{ij})-1\}$ , and finally we calculated the vector $Z_{i}$ such that $Z_{ij}=\Phi^{-1}(U_{ij})$ , where Φ(⋅) is the standard normal cumulative distribution function. Following,^29^ $Z_{i}$ has a multivariate chi-square copula distribution with a non-centrality parameter a≥0 , and normal marginal distributions. In our simulations, we fixed a=1 .

In all three settings, we considered $h^{2}\in \{0,0.2,0.5\}$ . Table 1 describes the parameters’ combinations used in our simulation studies.

**Table 1.** Parameters combinations for the simulations studies under all the settings and scenarios I: family size; N: total of sample size; $h^{2}:$ polygenic heritability; τ: variance component (VC); $\gamma_{0}:$ intercept; $\gamma_{1}\;\text{and}\;\gamma_{2}:$ covariate effects; β: genotypes effects; $X_{1}\;\text{and}\;X_{2}:$ covariates.

Parameters	Values
I	120
N	600
$h^{2}$	{0,0.2,0.5}
τ	{0.05,0.2}
$\gamma_{0}$	−2
$\gamma_{1}$	1
$\gamma_{2}$	1
β	$N_{20}(0,0)$ (under null) — $\;N_{20}(0,\tau W_{20})$ (under alternative)
$X_{1}$	U[0,1]
$X_{2}$	discrete 0/1 with probability of succes 0.2

Under the null model, the parameter of interest is $\beta =0_{r}$ ; then B=10,000 random samples were generated according to each parameter-combination scenario to assess the type I error rate of all the methods. We again refer the reader to Table 1 for the specific values of the model parameter combination considered.

To evaluate the methods’ performance in terms of power, five SNPs were randomly chosen among the 20 studied SNPs as causal variants (i.e. 25% of all studied SNPs). More precisely, for the five causal SNPs, we assumed their effect $\beta_{causal}∼N_{5}(0,\tau W_{5})$ , with τ∈{0.05,0.2}, and $W_{5}=diag(w_{1},\ldots ,w_{5})$ is a 5×5 diagonal matrix of a priori weights to be used for the 5 variants. The effects of the remaining SNPs were set to zero. The power comparison was based on B=1000 replications.

### 3.2. Simulation results

This section summarizes the simulation results for the Type I error rate for the scenario with $h^{2}=0.5,$ and the power levels for all settings described above.

#### 3.2.1. Type I error results

**Figure 1.**
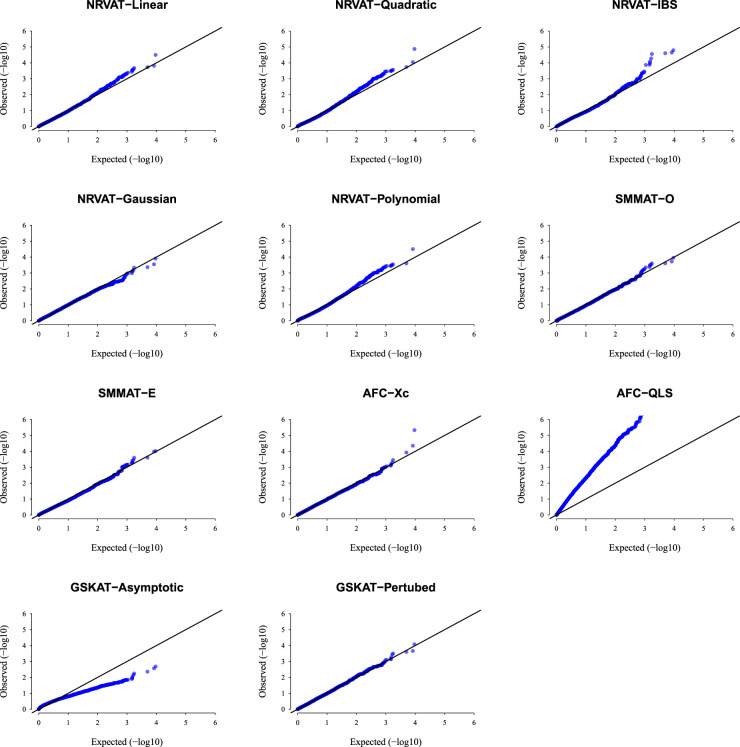
QQ-plot under the null hypothesis of no SNPs/phenotype association (τ=0), with the heritability parameter $h^{2}=0.5,$ where the data are generated under the G copula. Results are computed from 10,000 data sets generated under Setting 1. The compared methods are NRVAT model with the L, Q, IBS, G, and P kernel matrices; SMMAT model with the hybrid test (O), and the efficient hybrid test (E); AFC model with $X_{\text{c}}^{2}$ (Xc), and $W_{\text{QLS}}$ (QLS); and gSKAT model with the asymptotic and perturbed. QQ: quantile–quantile; SNP: single nucleotide polymorphism; NRVAT: novel rare variant association test; L: linear; Q: quadratic; IBS: identity-by-state; G: Gaussian; P: polynomial; SMMAT: variant-set mixed model association tests; AFC: allele frequency comparison; GSKAT: burden and kernel-based gene set association tests for binary traits.

*Results of Setting 1:* Figure 1 and Table S1 (Supplemental material) show quantile–quantile (QQ)-plots of the p -values and empirical type I error rate of all the considered methods, respectively, where data are generated under the Gaussian copula model. The empirical type I error of NRVAT, SMMAT, AFC (Xc) and gSKAT (perturbed) are around the nominal level ( α=0.01 ). By contrast, the empirical type I error of gSKAT (asymptotic) is lower than the nominal level whereas AFC_QLS has an inflated type I error rate. Our proposed approach showsa well-controlled type I error rate, for all the proposed Kernel-based tests. Figures S1 and S2 (Supplemental material) show similar results under $h^{2}\in \{0,0.2\},$ respectively.

**Figure 2.**
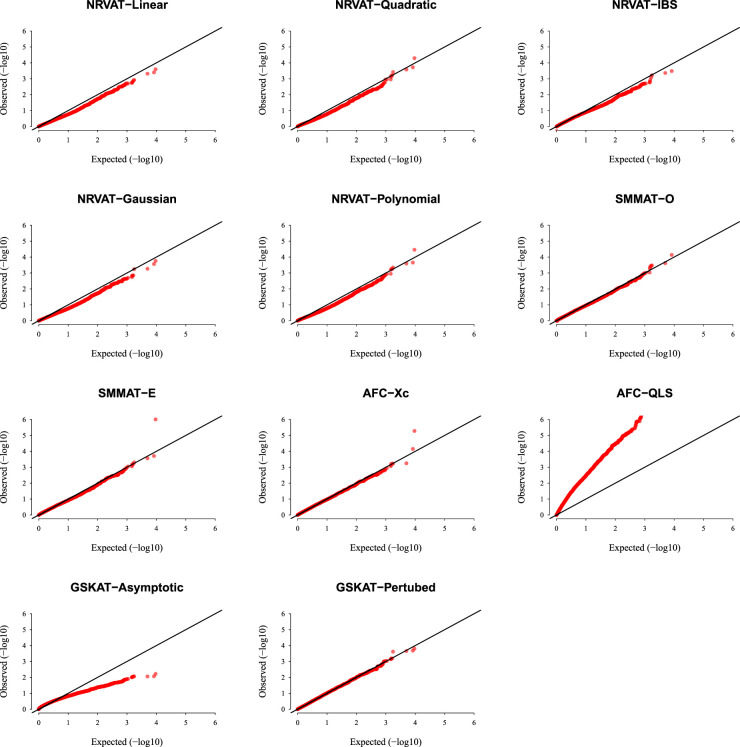
QQ-plot under the null hypothesis of no SNPs/phenotype association (τ=0), with the heritability parameter $h^{2}=0.5,$ where the data are generated under the GLMM. Results are computed from 10,000 data sets generated under Setting 2. The compared methods are NRVAT model with the L, Q, IBS, G, and P kernel matrices; SMMAT model with the hybrid test (O), and the efficient hybrid test (E); AFC model with $X_{\text{c}}^{2}$ (Xc), and $W_{\text{QLS}}$ (QLS); and GSKAT model with the asymptotic and perturbed. QQ: quantile–quantile; SNP: single nucleotide polymorphism; GLMM: generalized linear mixed model; NRVAT: novel rare variant association test; L: linear; Q: quadratic; IBS: identity-by-state; G: Gaussian; P: polynomial; SMMAT: variant-set mixed model association tests; AFC: allele frequency comparison; GSKAT: burden and kernel-based gene set association tests for binary traits.

*Results of Setting 2:* Figure 2 and Table S2 (Supplemental material) show the results of all the methods when the data are generated from the GLMM model. As expected, the empirical type I error rate of SMMAT is well-controlled since it is a GLMM-based association test. The empirical type I error of NRVAT is controlled for $h^{2}\in \{0,0.2\}$ (Figures S3 and S4 of Supplemental material, respectively), however, the method seems to be conservative for $h^{2}=0.5.$ This might be due to the bias introduced in the NRVAT estimation of the marginal model parameters using the GLM model (1). In fact, from Table S6 of Supplemental material, one can see that the bias in the estimation of $\gamma_{0}$ , $\gamma_{1}$ , and $\gamma_{2}$ , increased as $h^{2}$ increased. On the other hand, AFC (Xc) and gSKAT (perturbed) show no type I error rate inflation, for the three values of the polygenic heritability ( $h^{2}$ ). The gSKAT (asymptotic) is still conservative whereas AFC test (QLS) remains inflated. Figures S3 and S4 of Supplemental material show similar results under $h^{2}\in \{0,0.2\},$ respectively.

**Figure 3.**
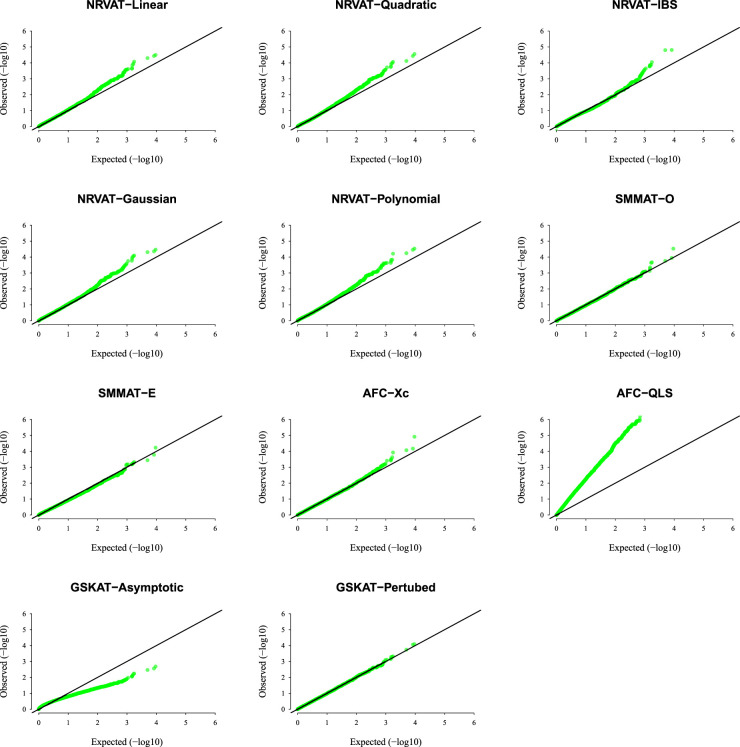
QQ-plot under the null hypothesis of no SNPs/phenotype association (τ=0), with the heritability parameter $h^{2}=0.5,$ where the data are generated under the student t copula model ( df=3 ). Results are computed from 10,000 data sets generated under Scenario 1 of Setting 3. The compared methods are NRVAT model with the L, Q, IBS, G, and P kernel matrices; SMMAT model with the hybrid test (O), and the efficient hybrid test (E); AFC model with $X_{\text{c}}^{2}$ (Xc), and $W_{\text{QLS}}$ (QLS); and GSKAT model with the asymptotic and perturbed. QQ: quantile–quantile; SNP: single nucleotide polymorphism; NRVAT: novel rare variant association test; L: linear; Q: quadratic; IBS: identity-by-state; G: Gaussian; P: polynomial; SMMAT: variant-set mixed model association tests; AFC: allele frequency comparison; GSKAT: burden and kernel-based gene set association tests for binary traits.

**Figure 4.**
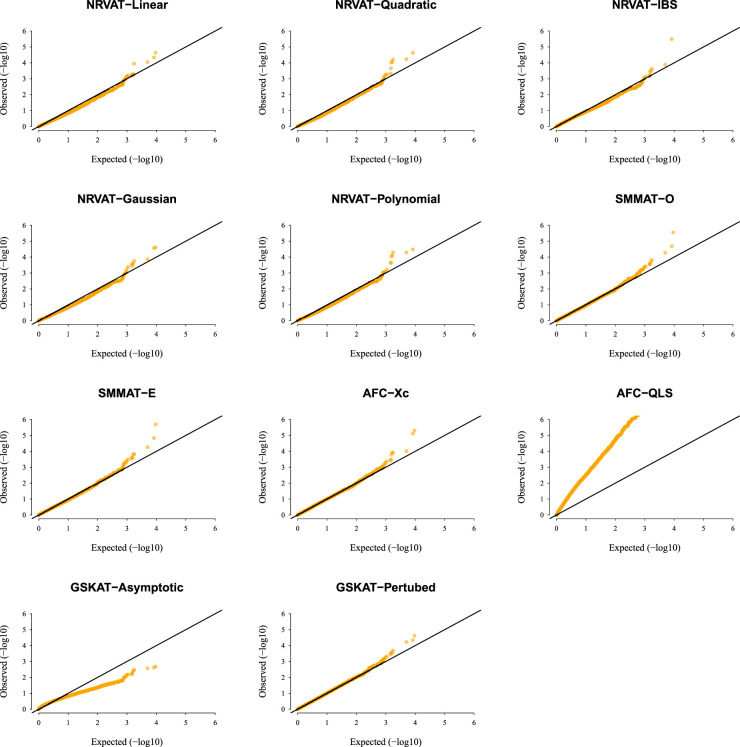
QQ-plot under the null hypothesis of no SNPs/phenotype association (τ=0), with the heritability parameter $h^{2}=0.5,$ where the data are generated under the chi-square copula model, with a non-centrality parameter a=1 . Results are computed from 10,000 data sets generated under Scenario 2 of Setting 3. The compared methods are NRVAT model with the L, Q, IBS, G, and P kernel matrices; SMMAT model with the hybrid test (O), and the efficient hybrid test (E); AFC model with $X_{\text{c}}^{2}$ (Xc), and $W_{\text{QLS}}$ (QLS); and GSKAT model with the asymptotic and perturbed. QQ: quantile–quantile; SNP: single nucleotide polymorphism; NRVAT: novel rare variant association test; L: linear; Q: quadratic; IBS: identity-by-state; G: Gaussian; P: polynomial; SMMAT: variant-set mixed model association tests; AFC: allele frequency comparison; GSKAT: burden and kernel-based gene set association tests for binary traits.

*Results of Setting 3:* Figure 3 and Table S3 (Supplemental material), and Figure 4 and Table S4 (Supplemental material) describe the empirical type I error rate results for data generated under the student t and the chi-square copula models, respectively. Under the student t copula model (Figures 3 and SupplementalTable S3), NRVAT seems to be sensitive to the true copula miss-specification by showing a slightly higher empirical type I error rate compared to the nominal level α=0.01 . However, under the chi-square copula model (Figures 4 and Supplemental Table S4), NRVAT shows a well-controlled type I error. On the contrary, in both scenarios of Setting 3, all the competitors behave in a similar way as in Setting 1. That is, SMMAT, AFC (Xc) and gSKAT (perturbed) have valid type I error rates, whereas AFC test (QLS) remains inflated and gSKAT (asymptotic) remains conservative. Figures S5 to S8 show similar results under $h^{2}\in \{0,0.2\},$ for the two scenarios of Setting 3, respectively.

We also show in Supplemental Tables S7 and S8, the results of empirical bias, for our method, of the nuisance parameters and the polygenic heritability under $H_{0}$ for the two scenarios of Setting 3.

Of note, the proposed method, like its competitors, might be sensitive to selection bias and it does not handle missing values; that is, subjects with missing values are removed from the data analysis. Although this is beyond the scope of this work, because real familial data are subject to selection bias and/or missing genotypes/phenotypes, we have conducted a sensitivity analysis to emphasize the impact of selection bias and missing data on our method. In Supplemental materials, we also show the conducted additional simulations under the null hypothesis for *Setting 1*. The simulated data were generated under three scenarios : (a) selection bias, (b) missing at random, and (c) missing completely at random.

To emphasize the impact of linkage disequilibrium between SNPs (i.e. $d^{2}$ values) on the performance of the proposed method and its competitors, we have carried out additional simulation scenarios with different values of $d^{2}$ . More precisely, in Supplemental materials, we presented additional simulations under the null hypothesis of *Setting 1* where the simulated data were generated under two scenarios: (1) $d^{2}=0.25$ , and (2) $d^{2}=0.36$ .

#### 3.2.2. Power results

**Figure 5.**
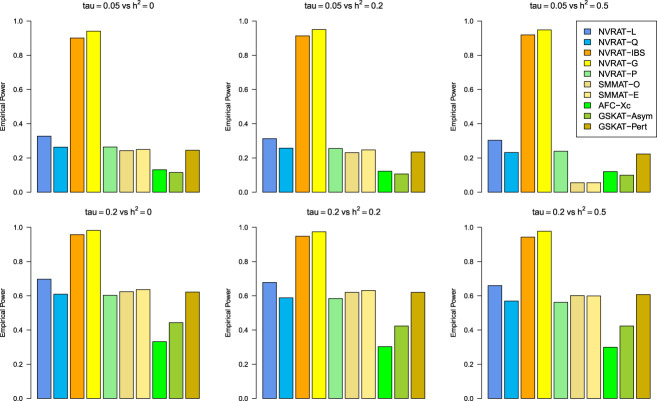
Empirical power under the alternative hypothesis of SNPs/phenotype association τ=0.05 and τ=0.2 (respectively, for the first line and the second line) where the data are generated under the Gaussian copula. Results are computed from 1000 data sets generated with 25% of causal variants taken randomly from the regions’ size (20) under Setting 1. The compared methods are NRVAT model with the L, Q, IBS, G, and P kernel matrices; SMMAT model with the hybrid test (O), and the efficient hybrid test (E); AFC model with $X_{\text{c}}^{2}$ (Xc); and GSKAT model with the asymptotic and perturbed. SNP: single nucleotide polymorphism; NRVAT: novel rare variant association test; L: linear; Q: quadratic; IBS: identity-by-state; G: Gaussian; P: polynomial; SMMAT: variant-set mixed model association tests; AFC: allele frequency comparison; GSKAT: burden and kernel-based gene set association tests for binary traits.

**Figure 6.**
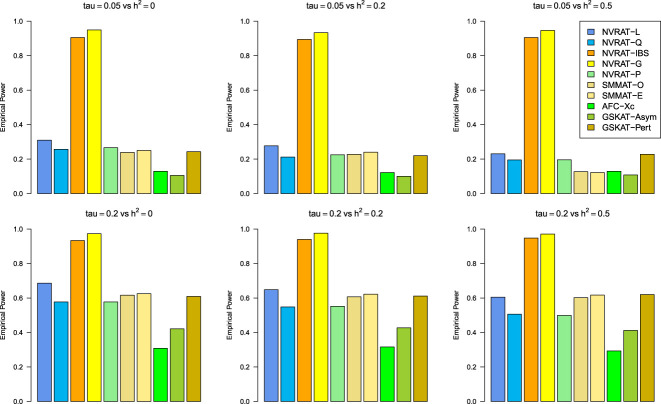
Empirical power under the alternative hypothesis of SNPs/phenotype association τ=0.05 and τ=0.2 (respectively, for the first line and the second line) where the data are generated under the GLMM. Results are computed from 1000 data sets generated with 25% of causal variants taken randomly from the regions size (20) under Setting 2. The compared methods are NRVAT model with the L, Q, IBS, G, and P kernel matrices; SMMAT model with the hybrid test (O), and the efficient hybrid test (E); AFC model with $X_{\text{c}}^{2}$ (Xc); and GSKAT model with the asymptotic and perturbed. SNP: single nucleotide polymorphism; GLMM: generalized linear mixed model; NRVAT: novel rare variant association test; L: linear; Q: quadratic; IBS: identity-by-state; G: Gaussian; P: polynomial; SMMAT: variant-set mixed model association tests; AFC: allele frequency comparison; GSKAT: burden and kernel-based gene set association tests for binary traits.

**Figure 7.**
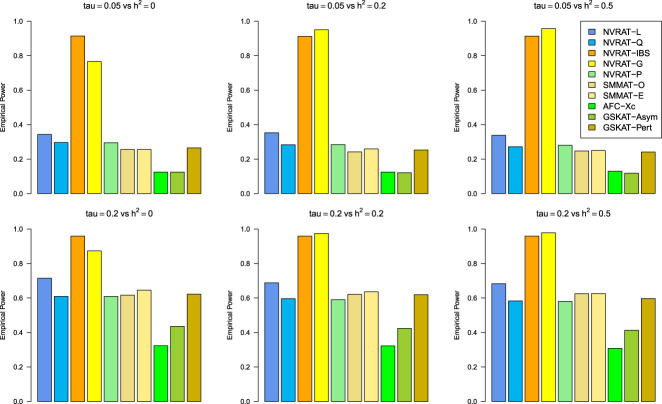
Empirical power under the alternative hypothesis of SNPs/phenotype association τ=0.05 and τ=0.2 (respectively, for the first line and the second line) where the data are generated under the student copula ( df=3 ). Results are computed from 1000 data sets generated with 25% of causal variants taken randomly from the regions size (20) under scenario 1 of Setting 3. The compared methods are NRVAT model with the L, Q, IBS, G, and P kernel matrices; SMMAT model with the hybrid test (O), and the efficient hybrid test (E); AFC model with $X_{\text{c}}^{2}$ (Xc); and GSKAT model with the asymptotic and perturbed. SNP: single nucleotide polymorphism; NRVAT: novel rare variant association test; L: linear; Q: quadratic; IBS: identity-by-state; G: Gaussian; P: polynomial; SMMAT: variant-set mixed model association tests; AFC: allele frequency comparison; GSKAT: burden and kernel-based gene set association tests for binary traits.

**Figure 8.**
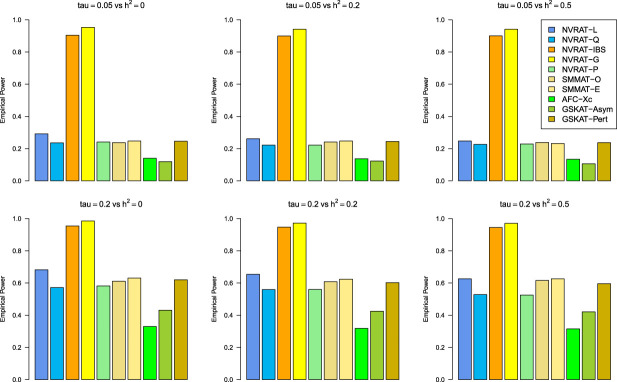
Empirical power under the alternative hypothesis of SNPs/phenotype association τ=0.05 and τ=0.2 (respectively, for the first line and the second line) where the data are generated under the chi-square copula with a non-centrality parameter a=1 . Results are computed from 1000 data sets generated with 25% of causal variants taken randomly from the regions’ size (20) under Scenario 2 of Setting 3. The compared methods are NRVAT model with the L, Q, IBS, G, and P kernel matrices; SMMAT model with the hybrid test (O), and the efficient hybrid test (E); AFC model with $X_{\text{c}}^{2}$ (Xc); and GSKAT model with the asymptotic and perturbed. SNP: single nucleotide polymorphism; NRVAT: novel rare variant association test; L: linear; Q: quadratic; IBS: identity-by-state; G: Gaussian; P: polynomial; SMMAT: variant-set mixed model association tests; AFC: allele frequency comparison; GSKAT: burden and kernel-based gene set association tests for binary traits.

**Figure 9.**
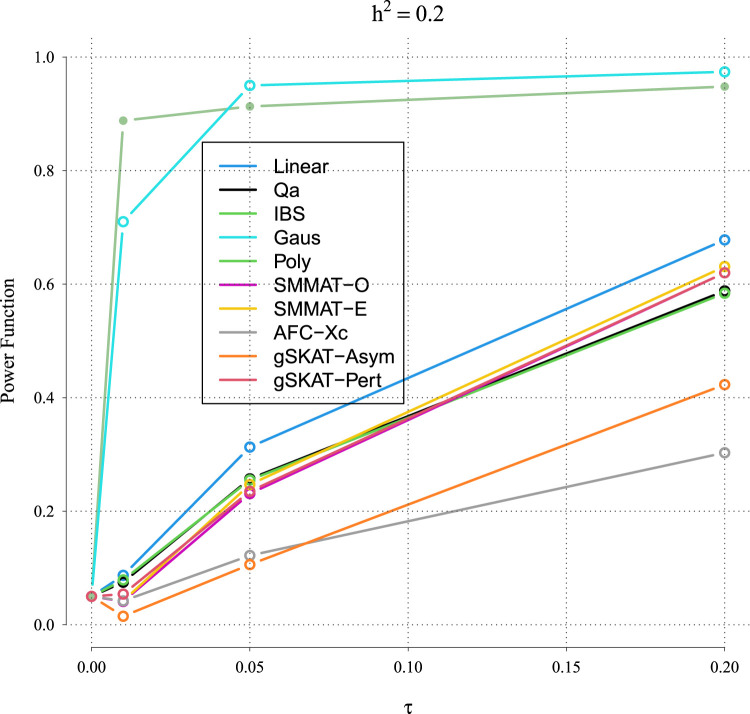
Power function under the alternative hypothesis of SNPs/phenotype association of grid of τ∈{0,0.01,0.05,0.2} for the polygenic heritability parameter $h^{2}=0.2$ where the data are generated under the Gaussian copula. Results are computed from 1000 data sets generated with 25% of causal variants taken randomly from the regions size (20) under Setting 1. The compared methods are NRVAT model with the L, Q, IBS, G, and P kernel matrices; SMMAT model with the hybrid test (O), and the efficient hybrid test (E); AFC model with $X_{\text{c}}^{2}$ (Xc); and gSKAT model with the asymptotic and perturbed. SNP: single nucleotide polymorphism; NRVAT: novel rare variant association test; L: linear; Q: quadratic; IBS: identity-by-state; G: Gaussian; P: polynomial; SMMAT: variant-set mixed model association tests; AFC: allele frequency comparison; GSKAT: burden and kernel-based gene set association tests for binary traits.

Figures 5 to 8 outline the empirical power levels for all the methods where data is generated from the Gaussian copula model (Setting 1), GLMM (Setting 2), and the student and chi-square copula models (Setting 3). The AFC (QLS) method was omitted in the power analysis comparison as it demonstrates severe type I error rate inflation (Figures 1 to 4 and Figures S1 to S8 of Supplemental material). Interestingly, in all settings, one can notice a substantial gain in the power of NRVAT with both the IBS and the Gaussian Kernel matrices. Figure 9 and Figures S9 and S10 of Supplemental material show the power levels as a function of a grid of values of the variance-component τ, respectively, for $h^{2}=0.2,$ $h^{2}=0$ and $h^{2}=0.5$ , under the Gaussian copula model (Setting 1). Again, these figures illustrate the important gain in power achieved by NRVAT with the IBS and the Gaussian Kernel similarity matrices.

## 4. Application to Real Data

### 4.1. Schizophrenia and bipolar disorder family study

The data in this analysis consists of 640 subjects belonging to 17 extended families from Eastern Quebec, with some family members known to have schizophrenia (SZ) or bipolar disorder (BP).^30^ We considered gene-based association analyses of SZ ( 61 affected; 435 non-affected and 144 unknown) and BP ( 91 affected; 405 non-affected and 144 unknown) binary phenotypes; we also analysed a ‘common locus’ (CL) binary phenotype, for which, diseased individuals are defined as subjects with SZ and/or BP ( 166 affected; 330 non-affected and 144 unknown). We considered genomics regions significant with linkage finding in SZ and BP from Quebec Eastern family data.^30^ That is, for the gene-based analysis of the SZ trait, 10,088 SNPs clustered within 291 genes were considered. The analysis of the BP trait considered 5979 SNPs falling within 163 genes. The CL phenotype analysis consisted of 9919 SNPs and 281 genes. The whole-genome SNP genotyping was provided by OmniExpress24 Illumina and the genotype data was prepared by Chagnon et al.^30^ After removing both subjects and SNPs with missing values, a total of 433 subjects were available for analysis, with 57 affected (AF) and 376 non-affected relatives (NAR) for SZ, 83 AF versus 350 NAR for BP, and 153 AF against 280 NAR for CL. Finally, 288 genes were retained for SZ analysis with a total of 10085 SNPs with gene size varying between 2 and 239 SNPs with 13% of SNPs having their MAF <5%. BP analysis has considered 161 genes for a total of 5977 SNPs, with gene-size varying from 2 to 285 SNPs with 12% of their SNPs have MAF <5%. The genotypes of the SNPs corresponding to the CL genomics region were all available, and so, there was no reduction in the number of SNPs; the gene size, in this analysis, ranges between 2 and 285 SNPs with 13% of SNPs having their MAF <5%. For the three traits, we fitted NRVAT, SMMAT, AFC, and gSKAT set-based association tests. In all analyses, we adjusted for the sex ( 255 females vs. 178 males) as a covariate in all fitted models.

### 4.2. Results of SZ and BP analysis

**Figure 10.**
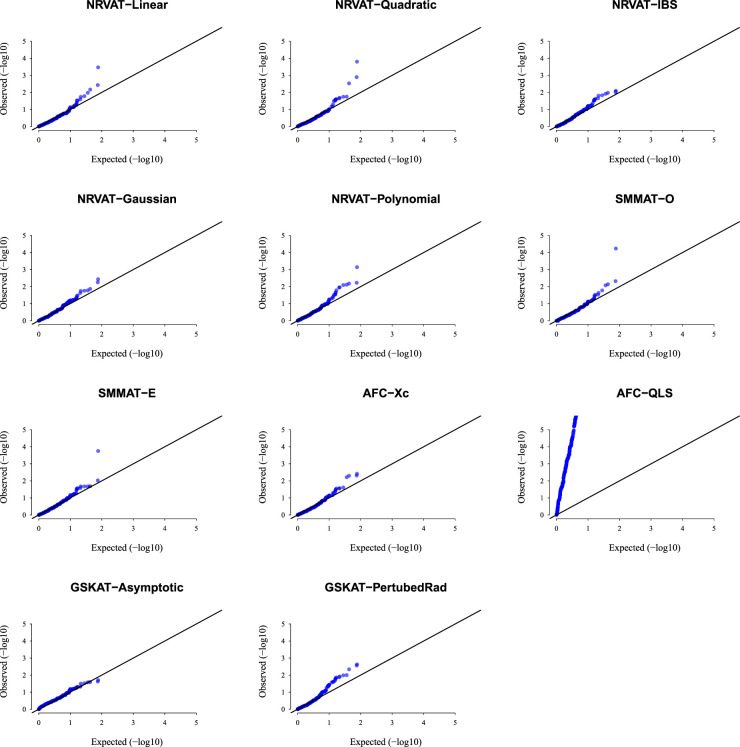
QQ-plot for NRVAT model with the L, Q, IBS, G, and P kernel matrices; SMMAT model with the hybrid test (O), and the efficient hybrid test (E); AFC model with $X_{\text{c}}^{2}$ (Xc), and $W_{\text{QLS}}$ (QLS); and GSKAT model with the asymptotic and perturbed for schizophrenia family study data. QQ: quantile–quantile; NRVAT: novel rare variant association test; L: linear; Q: quadratic; IBS: identity-by-state; G: Gaussian; P: polynomial; SMMAT: variant-set mixed model association tests; AFC: allele frequency comparison; GSKAT: burden and kernel-based gene set association tests for binary traits.

**Figure 11.**
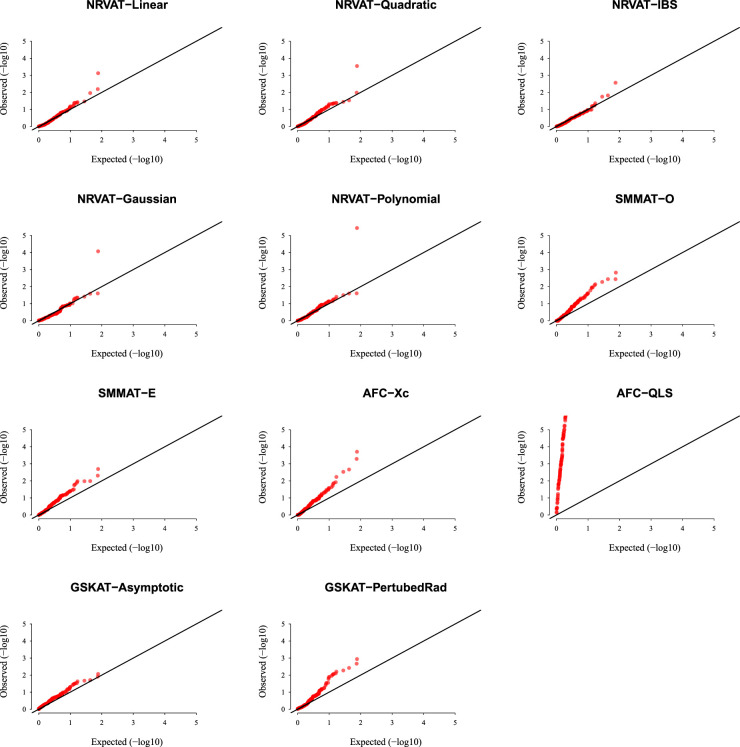
QQ-plot for NRVAT model with the L, Q, IBS, G, and P kernel matrices; SMMAT model with the hybrid test (O), and the efficient hybrid test (E); AFC model with $X_{\text{c}}^{2}$ (Xc), and $W_{\text{QLS}}$ (QLS); and GSKAT model with the asymptotic and perturbed for bipolar disorder family study data. QQ: quantile–quantile; NRVAT: novel rare variant association test; L: linear; Q: quadratic; IBS: identity-by-state; G: Gaussian; P: polynomial; SMMAT: variant-set mixed model association tests; AFC: allele frequency comparison; GSKAT: burden and kernel-based gene set association tests for binary traits.

**Figure 12.**
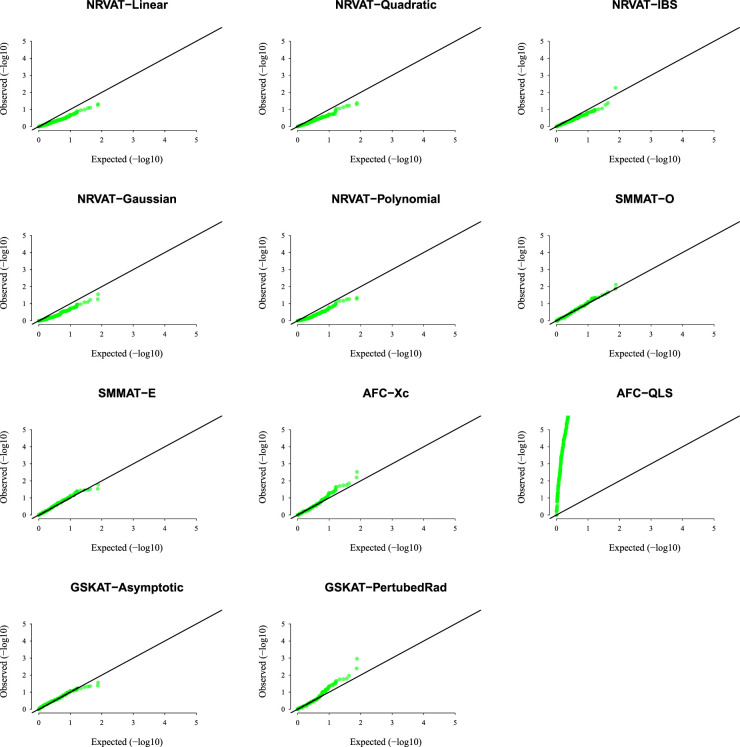
QQ-plot for NRVAT model with the L, Q, IBS, G, and P kernel matrices; SMMAT model with the hybrid test (O), and the efficient hybrid test (E); AFC model with $X_{\text{c}}^{2}$ (Xc), and $W_{\text{QLS}}$ (QLS); and GSKAT model with the asymptotic and perturbed for CL, for which, diseased individuals are defined as subjects with SZ and/or BP, family study data. QQ: quantile–quantile; NRVAT: novel rare variant association test; L: linear; Q: quadratic; IBS: identity-by-state; G: Gaussian; P: polynomial; SMMAT: variant-set mixed model association tests; AFC: allele frequency comparison tests: GSKAT: burden and kernel-based gene set association tests for binary traits; CL: common locus; SZ: schizophrenia; BP: bipolar disorder.

**Figure 13.**
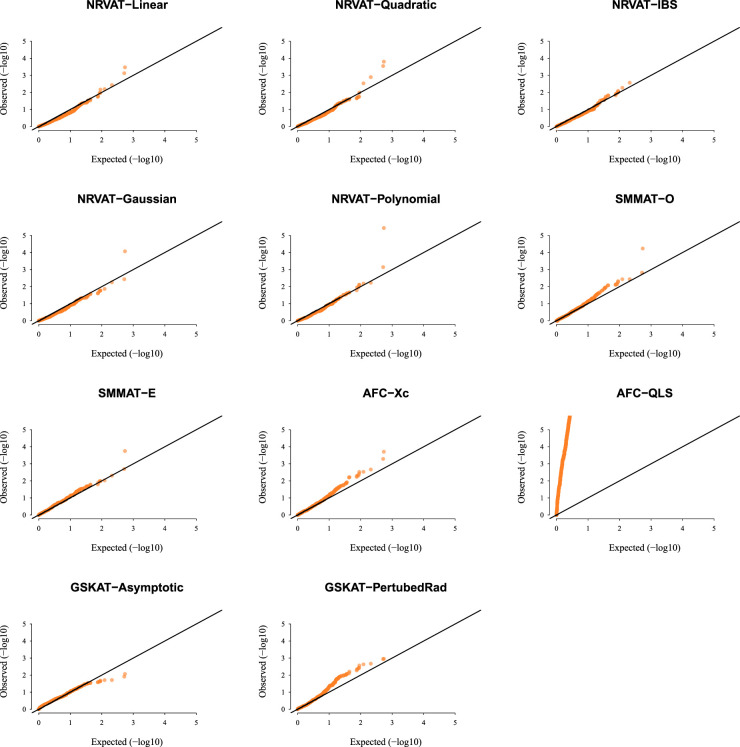
QQ-plot for NRVAT model with the L, Q, IBS, G, and P kernel matrices; SMMAT model with the hybrid test (O), and the efficient hybrid test (E); AFC model with $X_{\text{c}}^{2}$ (Xc), and $W_{\text{QLS}}$ (QLS); and GSKAT model with the asymptotic and perturbed for schizophrenia, bipolar disorder and their CL family study data for overall (combining SZ, BP and CL). QQ: quantile–quantile; NRVAT: novel rare variant association test; L: linear; Q: quadratic; IBS: identity-by-state; G: Gaussian; P: polynomial; SMMAT: variant-set mixed model association tests; AFC: allele frequency comparison tests: gSKAT: burden and kernel-based gene set association tests for binary traits; CL: common locus; SZ: schizophrenia; BP: bipolar disorder.

Figures 10 to 12 show the QQ plots of the obtained p -values from the gene-based analyses of SZ, BP, and CL. Figure 13 shows the overall p -value QQ-plot, in which, p -values from the analysis of the three traits are put together as one set of p -values. From these figures, one can see that all the methods have valid type I error rates under the analysis of both SZ and CL traits (Figures 10 and 12), except AFC (QLS), which has severe inflated type I error rate as it was also noticed in the simulation results. On the contrary, under the BP analysis, AFC (XC), SMMAT, and gSKAT (perturbed) present inflated type I error rate, however, NRVAT still has valid results in this analysis.

Table 2 reports genes/regions with p -values that are significant at the nominal significance level of 0.05 , after correcting for multiple testing using Bonferroni correction, for all the methods. Under SZ analysis, the strongest association is detected for gene *SMYD3* by our method with the quadratic kernel matrix (NRVAT-Quadratic) and the SMMAT model with the hybrid test (O) method ( p -value $\leq 0.05/288=1.74\times 10^{-4}$ ). Significant signals are also detected, under the BP analysis, for the genes *C1ORF77*, *CGI-96*, and *TRIM24* for NRVAT-Quadratic, NRVAT-Gaussian, NRVAT-Polynomial, and AFC (Xc) ( p -value $\leq 0.05/161=3.1\times 10^{-4}$ ). No significant signals were declared from the CL analysis.

**Table 2.** Significant genes after Bonferroni correction of NRVAT model with the L, Q, IBS, G, and P kernel matrices; SMMAT model with the hybrid test (O), and the efficient hybrid test (E); AFC model with $X_{\text{corrected}}^{2}$ (Xc), and $W_{\text{QLS}}$ (QLS); and GSKAT model with the asymptotic and perturbed for SZ, BP disorder, and their CL family study data at nominal level α=0.05 and the overall Bonferroni correction, which corrects for multiple testing of the total number of tests conducted in the three analyses, for each method.

		NRVAT					SMMAT		AFC		GSKAT	
α	Traits	L	Q	IBS	G	P	O	E	Xc	QLS	Asymp	Pert
0.05	SZ	-	SMYD3 ( $16\times 10^{-05}$ )	–	–	–	SMYD3 ( $5.82\times 10^{-05}$ )	–	–	*	–	–
	BP	-	C1ORF77 ( $28\times 10^{-05}$ )	-	CGI-96 ( $8\times 10^{-05}$ )	CGI-96 ( $3.64\times 10^{-06}$ )	–	–	TRIM24 ( $19\times 10^{-05}$ )	*	–	–
	CL	–	–	–	–	–	–	–	–	*	–	–
	OverAll	–	–	–	–	CGI-96	SMYD3	–	–	*	–	–

NRVAT: novel rare variant association test; L: linear; Q: quadratic; IBS: identity-by-state; G: Gaussian; P: polynomial; CL: common locus; SMMAT: variant-set mixed model association tests; AFC tests: allele frequency comparison tests; Gskat: burden and kernel-based gene set association tests for binary traits; BP: bipolar disorder; SZ: schizophrenia; *: method with inflated type I error rate; –: non-significative methods.

Table 2 highlights also the significant results from the overall Bonferroni correction, which corrects for multiple testing of the total number of tests conducted in the three analyses, for each method; that is, Bonferroni-corrected threshold equals $0.05/(288+161+281)=6.85\times 10^{-5}$ . We note that the genes *CGI-96* and *SMYD3* remain significant for NRVAT-Polynomial and SMMAT-O methods, respectively. Indeed, *SMYD3* is one of the histone methyltransferases that catalyse methylation of histone H3 at K4 in mammalian cells,^31^ and it has been proven that one of it rare SNV namely rs6426297 is associated with the SZ trait. This rare SNVis associated with suicide attempts in people with SZ and BP (for more details, see https://www.ebi.ac.uk/gwas/variants/rs6426297).

## 5. Discussion

In this work, we have developed NRVAT, a flexible set-based association test for rare and common variants and binary phenotypes, in family-based designs. NRVAT uses marginal generalized linear mixed models to relate the outcome to both the covariates and a set of SNPs and uses copulas to account for possible dependence between subjects of the same family/pedigree through the kinship matrix. An advantage of NRVAT joint modelling is that the regression parameters linking both covariates and genotypes to the phenotype are marginally meaningful. Moreover, trait polygenic heritability is ‘margin-free’, in the sense that it is characterized by the copula alone. The NRVAT framework includes five different kernel matrices to capture genotype–phenotype relationships, namely, the linear (L), quadratic (Q), identity-by-state (IBS), Gaussian (G), and polynomial (P) kernel matrices. Through simulations, we have shown that NRVAT has a valid type I error rate and allows for more power than existing models in family-based designs, even when the true copula was not well specified. We have also evaluated the performance of the NRVAT model using schizophrenia and bipolar disorder data and we have found an association signal with the SZ binary trait for one gene *SMYD3*, and with the BP binary trait for two genes, *C1ORF77* and *CGI-96*.

One of the main advantages of NRVAT is that it models the dependence structure between subjects regardless of their marginal distributions, which allows the use of different margins to link the binary response to the covariates and the genotypes. For instance, one can use latent Probit or Robit^22^ marginal models instead of the latent logistic model in (1). Our method is also valid in the presence of selection bias or missing genotypes.

It is necessary to note that the NRVAT model has some limitations. In fact, the derivation of p -values and the asymptotic null distribution of the NRVAT score test statistic assumes that the families are independent. This is true when one uses a priori kinship matrix to describe subjects’ relatedness, which is a block diagonal matrix. However, in the absence of pedigree information and/or in the presence of admixture population effects, the estimation of subjects’ relatedness is more suitable using an empirical genetic similarity matrix, such as the GRM.^34,32,33^ Such matrices, by construction, are not block diagonals and thus their use within NRVAT might not be suitable. This means that NRVAT might not handle a population structure other than the well-defined familial structure. Also, people should be aware that the NRVAT model could give incorrect results when the number of families is small. Several extensions to our methodological proposal could also be investigated. Our approach could be extended to develop a functional association test for dichotomous traits in the presence of family data. To parallel what is done in Jiang et al.,^35^ genotype–phenotype functional relationship can be modelled in the marginal distributions based on generalized functional linear mixed models while, again, a copula model can be used to characterize the trait dependence between subjects of the same family. So far, our approach only handles a single binary trait for each subject, but an extension to a mixture of binary-continuous trait cases for familial data could be possible. In fact, an additional source of dependency, stemming from within-subject correlated phenotypic values, could be accounted for by choosing an appropriate copula model.

NRVAT integrates five kernel matrices within the association test to better capture the phenotype–genotype relationship. Although the optimal choice of the kernel matrix for real data analysis is a daunting question, here are some guidelines on how to choose a kernel matrix in NRVAT: when the underlying genotype–phenotype relationship is unknown, one may choose a linear kernel if there is a priori knowledge that relationships are linear and there are no interactions. In the presence of interactions, the quadratic kernel can be a good alternative as it implicitly assumes that the underlying relationship depends on the main and second-order effects.^4^ In the case where there is a priori knowledge of the existence of more complex relationships, the Gaussian and IBS kernels may be the better choice.

## Software availability

The NRVAT method is implemented in a software R package PedGFLMM, which is freely available at https://github.com/houssoudossa/NRVATGFLMM.

Codes are also publicly available in Github for reproducibility of the simulation scenarios of Setting 1 at https://github.com/houssoudossa/NRVAT-simulation-code.

## Supplemental Material

sj-pdf-1-smm-10.1177_09622802231197977 - Supplemental material for A novel rare variants association test for binary traits in family-based designs via copulas Click here for additional data file. Supplemental material, sj-pdf-1-smm-10.1177_09622802231197977 for A novel rare variants association test for binary traits in family-based designs via copulas by Houssou R. G. Dossa, Alexandre Bureau, Michel Maziade, Lajmi Lakhal-Chaieb and Karim Oualkacha in Statistical Methods in Medical Research

## Appendix

### Estimation of the covariates’ parameters under the null hypothesis

Under independence assumption, for i,…,I families with $n_{i}$ the size of the ith family, the marginal likelihood is as follow:

$$\begin{array}{rl} \ell_{ind} & =\prod_{i=1}^{I}\;\prod_{j=1}^{n_{i}}\;\ell_{ij}\\ & =\prod_{i=1}^{I}\;\prod_{j=1}^{n_{i}}\;P(Y_{ij}=y_{ij}|X_{1ij},\;X_{2ij})\\ & =\prod_{i=1}^{I}\;\prod_{j=1}^{n_{i}}\;\left\{\frac{e^{\;y_{ij}\;(\gamma_{0}+\gamma_{1}^{⊤}X_{1ij}+\gamma_{2}^{⊤}X_{2ij})}}{1+e^{\gamma_{0}+\gamma_{1}^{⊤}X_{1ij}+\gamma_{2}^{⊤}X_{2ij}}}\right\}. \end{array}$$

The log-likelihood ( $\log\;\ell_{ind}=l$ ) is given by

$$\begin{array}{rl} l & =\sum_{i=1}^{I}\;\sum_{j=1}^{n_{i}}\;\log\;\ell_{ij}\\ & =\sum_{i=1}^{I}\;\sum_{j=1}^{n_{i}}\;\log\;\left\{\frac{e^{\;y_{ij}\;(\gamma_{0}+\gamma_{1}^{⊤}X_{1ij}+\gamma_{2}^{⊤}X_{2ij})}}{1+e^{\left(\gamma_{0}+\gamma_{1}^{⊤}X_{1ij}+\gamma_{2}^{⊤}X_{2ij}\right)}}\right\}\\ & =\sum_{i=1}^{I}\;\sum_{j=1}^{n_{i}}\;\left\{y_{ij}\;(\gamma_{0}+\gamma_{1}^{⊤}X_{1ij}+\gamma_{2}^{⊤}X_{2ij})-\log\;(1+e^{\gamma_{0}+\gamma_{1}^{⊤}X_{1ij}+\gamma_{2}^{⊤}X_{2ij}})\right\}. \end{array}$$

The first partial derivative of the log-likelihood with respect to $\gamma =\left[\begin{array}{c} \gamma_{0}\\ \gamma_{1}\\ \gamma_{2} \end{array}\right]$ takes the form

$$\begin{array}{rl} \frac{\partial l}{\partial \gamma } & =\frac{\partial [\sum_{i=1}^{I}\;\sum_{j=1}^{n_{i}}\;y_{ij}\;(\gamma_{0}+\gamma_{1}^{⊤}X_{1ij}+\gamma_{2}^{⊤}X_{2ij})-\log\;(1+e^{\gamma_{0}+\gamma_{1}^{⊤}X_{1ij}+\gamma_{2}^{⊤}X_{2ij}})]}{\partial \gamma }\\ & =\sum_{i=1}^{I}\;\sum_{j=1}^{n_{i}}\;\left\{\frac{\partial [y_{ij}\;(\gamma_{0}+\gamma_{1}^{⊤}X_{1ij}+\gamma_{2}^{⊤}X_{2ij})]}{\partial \gamma }-\frac{\partial [\log\;(1+e^{\gamma_{0}+\gamma_{1}^{⊤}X_{1ij}+\gamma_{2}^{⊤}X_{2ij}})]}{\partial \gamma }\right\}\\ & =\sum_{i=1}^{I}\;\sum_{j=1}^{n_{i}}\;\left\{y_{ij}\left[\begin{array}{c} 1\\ X_{1ij}\\ X_{2ij} \end{array}\right]-\frac{e^{\gamma_{0}+\gamma_{1}^{⊤}X_{1ij}+\gamma_{2}^{⊤}X_{2ij}}\;}{1+e^{\gamma_{0}+\gamma_{1}^{⊤}X_{1ij}+\gamma_{2}^{⊤}X_{2ij}}}\left[\begin{array}{c} 1\\ X_{1ij}\\ X_{2ij} \end{array}\right]\right\}\\ & =\sum_{i=1}^{I}\;\sum_{j=1}^{n_{i}}\;\left\{(y_{ij}-\frac{e^{\gamma_{0}+\gamma_{1}^{⊤}X_{1ij}+\gamma_{2}^{⊤}X_{2ij}}}{1+e^{\gamma_{0}+\gamma_{1}^{⊤}X_{1ij}+\gamma_{2}^{⊤}X_{2ij}}})\left[\begin{array}{c} 1\\ X_{1ij}\\ X_{2ij} \end{array}\right]\right\}\\ & =\sum_{i=1}^{I}\;\sum_{j=1}^{n_{i}}\;(y_{ij}-\mu_{ij})\left[\begin{array}{c} 1\\ X_{1ij}\\ X_{2ij} \end{array}\right]\\ & =\sum_{i=1}^{I}\;S_{i}(\gamma )\\ & =S(\gamma ), \end{array}$$

with $S_{i}(\gamma )=\sum_{j=1}^{n_{i}}\;(y_{ij}-\mu_{ij})\left[\begin{array}{c} 1\\ X_{1ij}\\ X_{2ij} \end{array}\right]$ et $\mu_{ij}=\frac{e^{\;(\gamma_{0}+\gamma_{1}^{⊤}X_{1ij}+\gamma_{2}^{⊤}X_{2ij})}}{1+e^{\;(\gamma_{0}+\gamma_{1}^{⊤}X_{1ij}+\gamma_{2}^{⊤}X_{2ij})}}.$

The estimation of $\gamma =\left[\begin{array}{c} \gamma_{0}\\ \gamma_{1}\\ \gamma_{2} \end{array}\right]$ is obtained throught GLM.

Since $\hat{\gamma }$ is solution of $S(\gamma )=\sum_{i=1}^{I}\;S_{i}(\gamma )=0,$ it follows that

Using Taylor’s expansion of order 1 around γ, we got

$$0=S(\hat{\gamma })=S(\gamma )+\frac{\partial \;S(\gamma )}{\partial \gamma }(\hat{\gamma }-\gamma )$$

So,

$$\begin{array}{rl} \left(\hat{\gamma }-\gamma )=-{\left[\frac{\partial S(\gamma )}{\partial \gamma }\right]}^{-1}S(\gamma )\;⟹\;\sqrt{I}(\hat{\gamma }-\gamma \right) & =-{\left[\frac{1}{I}\frac{\partial S(\gamma )}{\partial \gamma }\right]}^{-1}\frac{1}{\sqrt{I}}S(\gamma )\\ & =-{\left[\frac{1}{I}\sum_{i=1}^{I}\frac{\partial }{\partial \gamma }S_{i}(\gamma )\right]}^{-1}\frac{1}{\sqrt{I}}\sum_{i=1}^{I}S_{i}(\gamma ) \end{array}$$

According to Slustky’s theorem, one can easily show that

I(γ^−γ)→Np(0,A−1B[A−1]⊤)(Normalit\'{e} Asymptotique)

where

$$A=-E\left[\frac{1}{I}\sum_{i=1}^{I}\frac{\partial }{\partial \gamma }S_{i}(\gamma )\right]=-E\left[\frac{1}{I}\frac{\partial }{\partial \gamma }S(\gamma )\right]\;\text{and}\;B=Var\left[\frac{1}{\sqrt{I}}\sum_{i=1}^{I}\;S_{i}(\gamma )\right]=\frac{1}{I}\sum_{i=1}^{I}Var[S_{i}(\gamma )]$$

Note that B is written quite easily. Indeed, by posing that

$$Y_{i}=\left[\begin{array}{c} y_{i1}\\ \vdots \\ y_{in_{i}} \end{array}\right],\;\mu_{i}=\left[\begin{array}{c} \mu_{i1}\\ \vdots \\ \mu_{in_{i}} \end{array}\right],\;X_{i}={\left[\begin{array}{ccc} 1 & X_{1i1} & X_{2i1}\\ \vdots & \vdots & \vdots \\ 1 & X_{1in_{i}} & X_{2in_{i}} \end{array}\right]}_{\left(n_{i}\times 3\right)}$$

we obtain

$$S_{i}(\gamma )=\sum_{j=1}^{n_{i}}\;(y_{ij}-\mu_{ij})\left[\begin{array}{c} 1\\ X_{1ij}\\ X_{2ij} \end{array}\right]=X_{i}^{⊤}(Y_{i}-\mu_{i})$$

so,

Hence

$$B=\frac{1}{I}\sum_{i=1}^{I}X_{i}^{⊤}Var(Y_{i})X_{i}$$

#### Estimation of the copula parameter $h^{2}$ under the null hypothesis

Once the parameters of the covariates are estimated, we proceed to estimate the parameter of the copula which turns out to be the estimate of heritability $h^{2}.$ To achieve this, we use the **plug-in** technique by replacing the estimates of the covariates’ parameters in the full marginal likelihood. This complete likelihood, although having an explicit form, can turn out to be complex in computational terms. To overcome this problem, we choose to proceed with the marginal composite likelihood method. Although the latter is designed to be much faster to calculate and it can be more robust for model specification errors, it can, however, be less statistically efficient.^36^

The marginal composite log-likelihood takes the form:

$$\begin{array}{rl} l_{i}(\hat{\gamma },h^{2}) & =\log\;\left[\prod_{i=1}^{I}\;\prod_{j\neq k}\;P(Y_{ij},Y_{ik})\right]\\ & =\sum_{i=1}^{I}\;\sum_{j\neq k}\;\log\;[P(Y_{ij},Y_{ik})] \end{array}$$

where $P(Y_{ij},Y_{ik})$ is the function composed by taking individuals two by two at the level of each family.

Four ( 04 ) possibilities can be taken into account for $P(Y_{ij},Y_{ik}).$ Indeed

$$P(Y_{ij},Y_{ik}|X)=\left\{ \begin{array}{l} P(Y_{ij}=1,Y_{ik}=1|X{)}^{Y_{ij}\times Y_{ik}}\\ P(Y_{ij}=0,Y_{ik}=1|X{)}^{(1-Y_{ij})\times Y_{ik}}\\ P(Y_{ij}=1,Y_{ik}=0|X{)}^{Y_{ij}\times (1-Y_{ik})}\\ P(Y_{ij}=0,Y_{ik}=0|X{)}^{\left(1-Y_{ij})\times (1-Y_{ik}\right)} \end{array}\right.$$

The explicit expression of $\log\;[P(Y_{ij},Y_{ik})]$ becomes

$$\begin{array}{rl} \log\;[P(Y_{i1},Y_{i2})] & =\left\{ \begin{array}{l} \left\{Y_{i1}\;Y_{i2}\}\log\;\{P(Y_{i1}=1,Y_{i2}=1|X)\right\}\\ \left\{(1-Y_{i1})\;Y_{i2}\}\log\;\{P(Y_{i1}=0,Y_{i2}=1|X)\right\}\\ \left\{Y_{i1}\;(1-Y_{i2})\}\log\;\{P(Y_{i1}=1,Y_{i2}=0|X)\right\}\\ \left\{\left(1-Y_{i1})\;(1-Y_{i2}\right)\}\log\;\{P(Y_{i1}=0,Y_{i2}=0|X)\right\} \end{array}\right.\\ & =\left\{ \begin{array}{l} \left\{Y_{i1}\;Y_{i2}\}\log\;\{C_{\Gamma }(\mu_{i1},\mu_{i2})\right\}\\ \left\{(1-Y_{i1})\;Y_{i2}\}\log\;\{\mu_{i2}-C_{\gamma }(\mu_{i1},\mu_{i2})\right\}\\ \left\{Y_{i1}\;(1-Y_{i2})\}\log\;\{\mu_{i1}-C_{\Gamma }(\mu_{i1},\mu_{i2})\right\}\\ \left\{\left(1-Y_{i1})\;(1-Y_{i2}\right)\}\log\;\{1-\mu_{i1}-\mu_{i2}+C_{\Gamma }(\mu_{i1},\mu_{i2})\right\} \end{array}\right. \end{array}$$

Thus, for each family, the log-likelihood is obtained by $l_{i}(\hat{\gamma },h^{2}).$ Once this log-likelihood has been defined, we proceed to the estimation of heritability ( $h^{2}$ ) using the technique **Optim**.

#### Proof

The proof of obtaining the four different possibilities of $P(Y_{ij},Y_{ik})$ is as follows.

Under $H_{0}:\beta =0$ which means $H_{0}:\tau =0,$ let suppose a bivariate copula $C_{\Gamma }({\bar{\mu }}_{ij},{\bar{\mu }}_{ik}{)}_{1\leq j,k\leq n_{i}}$ , with ${\bar{\mu }}_{ij}=1-\mu_{ij}.$

Let $P(Y_{ij}=1|X)=P(Z_{ij}\leq \Phi^{-1}(\mu_{ij}),\;\text{and }\;C_{\Gamma }(\mu_{ij},\mu_{ik})=P(Z_{ij}\leq \Phi^{-1}(\mu_{ij}),Z_{ik}\leq \Phi^{-1}(\mu_{ik})).$

$$\begin{array}{rl} P(Z_{ij}>\Phi^{-1}(\mu_{ij})) & =P(Z_{ij}>\Phi^{-1}(\mu_{ij}))\cap P(Z_{ik}>\Phi^{-1}(\mu_{ik})\cup Z_{ik}<\Phi^{-1}(\mu_{ik}))\\ & =P(Z_{ij}>\Phi^{-1}(\mu_{ij}),Z_{ik}>\Phi^{-1}(\mu_{ik}))+P(Z_{ij}>\Phi^{-1}(\mu_{ij}),Z_{ik}<\Phi^{-1}(\mu_{ik}))\\ & =P(Z_{ij}>\Phi^{-1}(\mu_{ij}),Z_{ik}>\Phi^{-1}(\mu_{ik}))+P(Z_{ik}<\Phi^{-1}(\mu_{ik}))-P(Z_{ij}\leq \Phi^{-1}(\mu_{ij}),Z_{ik}<\Phi^{-1}(\mu_{ik})) \end{array}$$

$$\begin{array}{rl} P(Z_{ij}\leq \Phi^{-1}(\mu_{ij})) & =P(Z_{ij}\leq \Phi^{-1}(\mu_{ij}))\cap P(Z_{ik}>\Phi^{-1}(\mu_{ik})\cup Z_{ik}<\Phi^{-1}(\mu_{ik}))\\ & =P(Z_{ij}\leq \Phi^{-1}(\mu_{ij}),Z_{ik}>\Phi^{-1}(\mu_{ik}))+P(Z_{ij}\leq \Phi^{-1}(\mu_{ij}),Z_{ik}<\Phi^{-1}(\mu_{ik})) \end{array}$$

- For $P(Y_{ij}=1,Y_{ik}=1|X),$
  $$\begin{array}{rl} P(Z_{ij}\leq \Phi^{-1}(\mu_{ij}),Z_{ik}<\Phi^{-1}(\mu_{ik})) & =P(Z_{ij}\leq \Phi^{-1}(\mu_{ij}),Z_{ik}\leq \Phi^{-1}(\mu_{ik}))\\ & =P(Z_{ij}>\Phi^{-1}(\mu_{ij}),Z_{ik}>\Phi^{-1}(\mu_{ik}))+P(Z_{ik}<\Phi^{-1}(\mu_{ik}))-P(Z_{ij}>\Phi^{-1}(\mu_{ij})\\ & =P(Z_{ij}\leq \Phi^{-1}(\mu_{ij}))+P(Z_{ik}\leq \Phi^{-1}(\mu_{ik}))-1+P(Z_{ij}>\Phi^{-1}(\mu_{ij}),Z_{ik}>\Phi^{-1}(\mu_{ik}))\\ & =\mu_{ij}+\mu_{ik}-1+C_{\Gamma }(1-\mu_{ij},1-\mu_{ik})\\ & =1-[1-\mu_{ij}]-[1-\mu_{ik}]+C_{\Gamma }(1-\mu_{ij},1-\mu_{ik})\\ & =C_{\Gamma }(\mu_{ij},\mu_{ik}) \end{array}$$
- For $P(Y_{ij}=0,Y_{ik}=1|X),$
  $$\begin{array}{rl} P(Z_{ij}>\Phi^{-1}(\mu_{ij}),Z_{ik}\leq \Phi^{-1}(\mu_{ik})) & =P(Z_{ij}>\Phi^{-1}(\mu_{ij})-P(Z_{ij}>\Phi^{-1}(\mu_{ij}),Z_{ik}>\Phi^{-1}(\mu_{ik}))\\ & =1-\mu_{ij}-C_{\Gamma }(1-\mu_{ij},1-\mu_{ik})\;\text{or}\\ & =P(Z_{ik}<\Phi^{-1}(\mu_{ik}))-P(Z_{ij}\leq \Phi^{-1}(\mu_{ij}),Z_{ik}\leq \Phi^{-1}(\mu_{ik}))\\ & =P(Z_{ik}\leq \Phi^{-1}(\mu_{ik}))-P(Z_{ij}\leq \Phi^{-1}(\mu_{ij}),Z_{ik}\leq \Phi^{-1}(\mu_{ik}))\\ & =\mu_{ik}-C_{\Gamma }(\mu_{ij},\mu_{ik}) \end{array}$$
- For $P(Y_{ij}=1,Y_{ik}=0|X),$
  $$\begin{array}{rl} & P(Z_{ij}\leq \Phi^{-1}(\mu_{ij}),Z_{ik}>\Phi^{-1}(\mu_{ik}))\\ & \;=P(Z_{ij}>\Phi^{-1}(\mu_{ij}))+P(Z_{ij}\leq \Phi^{-1}(\mu_{ij}))-P(Z_{ik}\leq \Phi^{-1}(\mu_{ik}))-P(Z_{ij}>\Phi^{-1}(\mu_{ij}),Z_{ik}>\Phi^{-1}(\mu_{ik}))\\ & \;=1-P(Z_{ij}\leq \Phi^{-1}(\mu_{ij}))+P(Z_{ij}\leq \Phi^{-1}(\mu_{ij}))-P(Z_{ik}\leq \Phi^{-1}(\mu_{ik}))-P(Z_{ij}>\Phi^{-1}(\mu_{ij}),Z_{ik}>\Phi^{-1}(\mu_{ik}))\\ & \;=1-P(Z_{ik}\leq \Phi^{-1}(\mu_{ik}))-P(Z_{ij}>\Phi^{-1}(\mu_{ij}),Z_{ik}>\Phi^{-1}(\mu_{ik}))\\ & \;=1-\mu_{ik}-C_{\Gamma }(1-\mu_{ij},1-\mu_{ik})\;\text{or}\\ & \;=P(Z_{ij}\leq \Phi^{-1}(\mu_{ij}))-P(Z_{ij}\leq \Phi^{-1}(\mu_{ij}),Z_{ik}<\Phi^{-1}(\mu_{ik}))\\ & \;=P(Z_{ij}\leq \Phi^{-1}(\mu_{ij}))-P(Z_{ij}\leq \Phi^{-1}(\mu_{ij}),Z_{ik}\leq \Phi^{-1}(\mu_{ik}))\\ & \;=\mu_{ij}-C_{\Gamma }(\mu_{ij},\mu_{ik}) \end{array}$$
- For $P(Y_{ij}=0,Y_{ik}=0|X),$
  $$\begin{array}{rl} P(Z_{ij}>\Phi^{-1}(\mu_{ij}),Z_{ik}>\Phi^{-1}(\mu_{ik})) & =P(Z_{ij}>\Phi^{-1}(\mu_{ij}))-P(Z_{ik}\leq \Phi^{-1}(\mu_{ik}))+P(Z_{ij}\leq \Phi^{-1}(\mu_{ij}),Z_{ik}<\Phi^{-1}(\mu_{ik}))\\ & =C_{\Gamma }(1-\mu_{ij},1-\mu_{ik})\;\text{or}\\ & =1-P(Z_{ij}\leq \Phi^{-1}(\mu_{ij}))-P(Z_{ik}\leq \Phi^{-1}(\mu_{ik}))+P(Z_{ij}\leq \Phi^{-1}(\mu_{ij}),Z_{ik}<\Phi^{-1}(\mu_{ik}))\\ & =1-P(Z_{ij}\leq \Phi^{-1}(\mu_{ij}))-P(Z_{ik}\leq \Phi^{-1}(\mu_{ik}))+P(Z_{ij}\leq \Phi^{-1}(\mu_{ij}),Z_{ik}\leq \Phi^{-1}(\mu_{ik}))\\ & =1-\mu_{ij}-\mu_{ik}+C_{\Gamma }(\mu_{ij},\mu_{ik}) \end{array}$$
  The above results confirm those found by Genest et al.^37^
  It therefore follows that
  $$P(Y_{ij},Y_{ik}|X)=\left\{ \begin{array}{l} \{1-[1-\mu_{ij}]-[1-\mu_{ik}]+C_{\Gamma }(1-\mu_{ij},1-\mu_{ik}){\}}^{Y_{ij}\times Y_{ik}}\\ \{1-\mu_{ij}-C_{\Gamma }(1-\mu_{ij},1-\mu_{ik}){\}}^{(1-Y_{ij})\times Y_{ik}}\\ \{1-\mu_{ik}-C_{\Gamma }(1-\mu_{ij},1-\mu_{ik}){\}}^{Y_{ij}\times (1-Y_{ik})}\\ \{C_{\Gamma }(1-\mu_{ij},1-\mu_{ik}){\}}^{\left(1-Y_{ij})\times (1-Y_{ik}\right)} \end{array}\right.$$
  with $\mu_{ij}=P(Y_{ij}=1|X)=\frac{e^{\gamma^{⊤}X_{ij}}}{1+e^{\gamma^{⊤}X_{ij}}}.$

## Appendix B

Let,

$$\begin{array}{rl} \gamma & =\left[\begin{array}{c} \gamma_{0}\\ \gamma_{1}\\ \gamma_{2} \end{array}\right],\;Y_{i}=\left[\begin{array}{c} y_{i1}\\ \vdots \\ y_{in_{i}} \end{array}\right],\;\mu_{i}=g_{i}(\gamma )=\left[\begin{array}{c} \mu_{i1}\\ \vdots \\ \mu_{in_{i}} \end{array}\right] \end{array}$$

(7)
$$\begin{array}{rl} \sqrt{I}(\hat{\gamma }-\gamma ) & =A^{-1}\;\frac{1}{\sqrt{I}}\sum_{k=1}^{I}\;S_{k}(\gamma )+o_{p}(1) \end{array}$$

(8)
$$\begin{array}{rl} S_{i}(\gamma ) & =X_{i}^{⊤}(Y_{i}-\mu_{i})=X_{i}^{⊤}[Y_{i}-g_{i}(\gamma )] \end{array}$$

under an extension of Taylor’s development of $g_{i}(\hat{\gamma })$ of order 1 around γ, we have that

(9)
$$g_{i}(\hat{\gamma })\approx g_{i}(\gamma )+\frac{\partial g_{i}(\gamma )}{\partial \gamma }(\hat{\gamma }-\gamma )$$

Suppose $D_{i}=\frac{\partial g_{i}(\gamma )}{\partial \gamma },$ the variance of the residuals becomes

$$\begin{array}{rl} Var(Y_{i}-{\hat{\mu }}_{i}) & =Var[Y_{i}-g_{i}(\hat{\gamma })]\\ & =E[(Y_{i}-g_{i}(\hat{\gamma }))(Y_{i}-g_{i}(\hat{\gamma }){)}^{⊤}]\\ & \approx E[(Y_{i}-g_{i}(\gamma )-D_{i}(\hat{\gamma }-\gamma ))(Y_{i}-g_{i}(\gamma )-D_{i}(\hat{\gamma }-\gamma ){)}^{⊤}]\\ & =E\{[(Y_{i}-g_{i}(\gamma ))-D_{i}(\hat{\gamma }-\gamma )][(Y_{i}-g_{i}(\gamma ))-D_{i}(\hat{\gamma }-\gamma ){]}^{⊤}\}\\ & =E[(Y_{i}-g_{i}(\gamma ))(Y_{i}-g_{i}(\gamma ){)}^{⊤}]-D_{i}E[(\hat{\gamma }-\gamma )(Y_{i}-g_{i}(\gamma ){)}^{⊤}]-E[(Y_{i}-g_{i}(\gamma ))(\hat{\gamma }-\gamma {)}^{⊤}]D_{i}^{⊤}\\ & \;+D_{i}E[(\hat{\gamma }-\gamma )(\hat{\gamma }-\gamma {)}^{⊤}]D_{i}^{⊤} \end{array}$$

From (7) and (8)

$$\begin{array}{rl} E[(\hat{\gamma }-\gamma )(Y_{i}-g_{i}(\gamma ){)}^{⊤}] & =E[(A^{-1}\;\frac{1}{I}\sum_{k=1}^{I}\;S_{k}(\gamma ))(Y_{i}-g_{i}(\gamma ){)}^{⊤}]\\ & =A^{-1}\;\frac{1}{I}\sum_{k=1}^{I}[X_{k}^{⊤}E\;[(Y_{k}-g_{k}(\gamma ))(Y_{i}-g_{i}(\gamma ){)}^{⊤}]]\\ & =A^{-1}\;\frac{1}{I}\;X_{i}^{⊤}E\;[(Y_{i}-g_{i}(\gamma ))(Y_{i}-g_{i}(\gamma ){)}^{⊤}] \end{array}$$

with

$$E\;[(Y_{k}-g_{k}(\gamma ))(Y_{i}-g_{i}(\gamma ){)}^{⊤}]=\left\{ \begin{array}{l} 0,\;\text{si}\;i\neq k\\ Var[Y_{i}-g_{i}(\gamma )],\;\text{sinon} \end{array}\right.$$

Then

and

So

$$\begin{array}{rl} Var(Y_{i}-{\hat{\mu }}_{i}) & =Var[Y_{i}-g_{i}(\hat{\gamma })]\\ & =Var[Y_{i}-g_{i}(\gamma )]-D_{i}A^{-1}\;\frac{1}{I}\;X_{i}^{⊤}Var[Y_{i}-g_{i}(\gamma )]-Var[Y_{i}-g_{i}(\gamma )]\;X_{i}\;\frac{1}{I}[A^{-1}{]}^{⊤}D_{i}^{⊤}+D_{i}E[(\hat{\gamma }-\gamma )(\hat{\gamma }-\gamma {)}^{⊤}]D_{i}^{⊤}\\ & =Var[Y_{i}-g_{i}(\gamma )]-D_{i}A^{-1}\;\frac{1}{I}\;X_{i}^{⊤}\;Var[Y_{i}-g_{i}(\gamma )]-Var[Y_{i}-g_{i}(\gamma )]\;X_{i}\;\frac{1}{I}[A^{-1}{]}^{⊤}D_{i}^{⊤}+D_{i}\;Var(\hat{\gamma }-\gamma )\;D_{i}^{⊤}\\ & =Var(Y_{i})-D_{i}A^{-1}\;\frac{1}{I}\;X_{i}^{⊤}\;Var(Y_{i})-Var(Y_{i})\;X_{i}\;\frac{1}{I}[A^{-1}{]}^{⊤}D_{i}^{⊤}+D_{i}\;A^{-1}\;B\;[A^{-1}{]}^{⊤}\;D_{i}^{⊤},\;(\text{under}\;H_{0})\\ & =\Sigma_{i}-D_{i}A^{-1}\;\frac{1}{I}\;X_{i}^{⊤}\;\Sigma_{i}-\Sigma_{i}\;X_{i}\;\frac{1}{I}[A^{-1}{]}^{⊤}D_{i}^{⊤}+D_{i}\;A^{-1}\;B\;[A^{-1}{]}^{⊤}\;D_{i}^{⊤} \end{array}$$

$$\begin{array}{rl} Cov[(Y_{i}-{\hat{\mu }}_{i})(Y_{k}-{\hat{\mu }}_{k})] & =E[(Y_{i}-{\hat{\mu }}_{i})(Y_{k}-{\hat{\mu }}_{k}{)}^{⊤}]-E[(Y_{i}-{\hat{\mu }}_{i})]E[(Y_{k}-{\hat{\mu }}_{k}{)}^{⊤}]\\ & =E[(Y_{i}-{\hat{\mu }}_{i})(Y_{k}-{\hat{\mu }}_{k}{)}^{⊤}]\\ & =E[(Y_{i}-g_{i}(\hat{\gamma }))(Y_{k}-g_{k}(\hat{\gamma }){)}^{⊤}]\\ & \approx E[(Y_{i}-g_{i}(\gamma )-D_{i}(\hat{\gamma }-\gamma ))(Y_{k}-g_{k}(\gamma )-D_{k}(\hat{\gamma }-\gamma ){)}^{⊤}]\\ & =E\{[(Y_{i}-g_{i}(\gamma ))-D_{i}(\hat{\gamma }-\gamma )][(Y_{k}-g_{k}(\gamma ))-D_{k}(\hat{\gamma }-\gamma ){]}^{⊤}\}\\ & =E[(Y_{i}-g_{i}(\gamma ))(Y_{k}-g_{k}(\gamma ){)}^{⊤}]-D_{i}E[(\hat{\gamma }-\gamma )(Y_{k}-g_{k}(\gamma ){)}^{⊤}]-E[(Y_{i}-g_{i}(\gamma ))(\hat{\gamma }-\gamma {)}^{⊤}]D_{k}^{⊤}\\ & \;+D_{i}E[(\hat{\gamma }-\gamma )(\hat{\gamma }-\gamma {)}^{⊤}]D_{k}^{⊤}\\ & =-D_{i}E[(\hat{\gamma }-\gamma )(Y_{k}-g_{k}(\gamma ){)}^{⊤}]-E[(Y_{i}-g_{i}(\gamma ))(\hat{\gamma }-\gamma {)}^{⊤}]D_{k}^{⊤}+D_{i}E[(\hat{\gamma }-\gamma )(\hat{\gamma }-\gamma {)}^{⊤}]D_{k}^{⊤}\\ & =-D_{i}A^{-1}\;\frac{1}{I}\;X_{i}^{⊤}\;Var[Y_{i}-g_{i}(\gamma )]-Var[Y_{k}-g_{k}(\gamma )]\;X_{k}\;\frac{1}{I}[A^{-1}{]}^{⊤}D_{k}^{⊤}+D_{i}\;Var(\hat{\gamma }-\gamma )\;D_{k}^{⊤}\\ & =-D_{i}A^{-1}\;\frac{1}{I}\;X_{i}^{⊤}\;Var(Y_{i})-Var(Y_{k})\;X_{k}\;\frac{1}{I}[A^{-1}{]}^{⊤}D_{k}^{⊤}+D_{i}\;A^{-1}\;B\;[A^{-1}{]}^{⊤}\;D_{k}^{⊤} \end{array}$$

Hence, one has

$$Cov[(Y_{i}-{\hat{\mu }}_{i})(Y_{k}-{\hat{\mu }}_{k})]=-\frac{1}{I}\;D_{i}A^{-1}\;X_{i}^{⊤}\;\Sigma_{i}-\frac{1}{I}\;\Sigma_{k}\;X_{k}\;[A^{-1}{]}^{⊤}D_{k}^{⊤}+\frac{1}{I}D_{i}\;A^{-1}\;B\;[A^{-1}{]}^{⊤}\;D_{k}^{⊤}$$

Since the families (i,k) are independents between themselves, we get

### Format of the estimated corrected variance-covariance matrix

The dimension of the estimated corrected variance–covariance matrix is of the form

- $\sum_{i=1}^{I}n_{i}\times n_{i}$ when the number of individuals differs from one family to another.
- nI×nI when the number of individuals is the same from one family to another.

In compact form, the estimated corrected variance–covariance matrix is written as follows:

$$\hat{\Omega }=\hat{\Sigma }-\frac{1}{I}M_{1}^{⊤}-\frac{1}{I}M_{1}+\frac{1}{I}M_{2}$$

where $M_{1}=\hat{\Sigma }\;X\;{\hat{A}}^{-1}\;{\hat{D}}^{⊤};\;$ $M_{2}=\hat{D}{\hat{A}}^{-1}\;\hat{B}{\hat{A}}^{-1}\;{\hat{D}}^{⊤};\;$ $\hat{\Sigma }=\text{Diag}\{{\hat{\Sigma }}_{i}{\}}_{i=1}^{I};\;$ $\hat{A}=X^{⊤}\;\hat{D}\;\text{and}\;$ $\hat{B}=X^{⊤}\hat{\Sigma }\;X,$

$\hat{D}=\left[\begin{array}{c} D_{1}\\ D_{2}\\ \vdots \\ D_{I} \end{array}\right]=\hat{\Delta }X$ ; $X=\left[\begin{array}{c} X_{1}\\ X_{2}\\ \vdots \\ X_{I} \end{array}\right]\;$ ; $\hat{\Delta }=\text{Diag}\{{\hat{\Delta }}_{i}{\}}_{i=1}^{I}\;$ and ${\hat{\Delta }}_{i}=\left[\begin{array}{cccc} {\hat{\mu }}_{i1}(1-{\hat{\mu }}_{i1}) & 0 & \ldots & 0\\ 0 & {\hat{\mu }}_{i2}(1-{\hat{\mu }}_{i2}) & \ldots & 0\\ \ldots & \ldots & \ldots & \ldots \\ 0 & 0 & \ldots & {\hat{\mu }}_{in_{i}}(1-{\hat{\mu }}_{in_{i}}) \end{array}\right]$ .

At the family level, the estimated corrected variance–covariance matrix can be written in the form

$${\hat{\Omega }}_{c}=Var_{c}(Y-\hat{\mu })=Var_{c}[(Y-\mu )-(\hat{\mu }-\mu )]=\left[\begin{array}{cccc} {\hat{\Omega }}_{1} & {\hat{\sigma }}_{12} & \ldots & {\hat{\sigma }}_{1I}\\ {\hat{\sigma }}_{12} & {\hat{\Omega }}_{2} & \ldots & {\hat{\sigma }}_{2I}\\ \ldots & \ldots & \ldots & \ldots \\ {\hat{\sigma }}_{I1} & {\hat{\sigma }}_{I2} & \ldots & {\hat{\Omega }}_{I} \end{array}\right]$$

where

$$\begin{array}{rl} {\hat{\Sigma }}_{i} & =\hat{Var}(Y_{i})\\ {\hat{\Omega }}_{i} & ={\hat{\Sigma }}_{i}-\frac{1}{I}\;{\hat{D}}_{i}{\hat{A}}^{-1}\;X_{i}^{⊤}\;{\hat{\Sigma }}_{i}-\frac{1}{I}\;{\hat{\Sigma }}_{i}\;X_{i}\;[{\hat{A}}^{-1}{]}^{⊤}{\hat{D}}_{i}^{⊤}+\frac{1}{I}{\hat{D}}_{i}\;{\hat{A}}^{-1}\;\hat{B}\;[{\hat{A}}^{-1}{]}^{⊤}\;{\hat{D}}_{i}^{⊤}\\ {\hat{\sigma }}_{ik} & =-\frac{1}{I}\;{\hat{D}}_{i}{\hat{A}}^{-1}\;X_{i}^{⊤}\;{\hat{\Sigma }}_{i}-\frac{1}{I}\;{\hat{\Sigma }}_{k}\;X_{k}\;[{\hat{A}}^{-1}{]}^{⊤}{\hat{D}}_{k}^{⊤}+\frac{1}{I}{\hat{D}}_{i}\;{\hat{A}}^{-1}\;\hat{B}\;[{\hat{A}}^{-1}{]}^{⊤}\;{\hat{D}}_{k}^{⊤} \end{array}$$
