# Supplementary material for: A novel rare variants association test for binary traits in family-based designs via copulas
Source: Stat Methods Med Res. 2023 Oct 13;32(11):2096–122. doi: 10.1177/09622802231197977 (PMC10683345; doi:10.1177/09622802231197977)
Supplement: sj-pdf-1-smm-10.1177_09622802231197977 - Supplemental material for A novel rare variants association test for binary traits in family-based designs via copulas [file sj-pdf-1-smm-10.1177_09622802231197977.pdf]

A Novel Rare Variants Association Test for Binary Traits in Family-Based Designs via Copulas

Houssou R. G. DOSSA [1], Alexandre Bureau[3], Michel Maziade[4], Lajmi Lakhal-Chaieb[2], and Karim Oualkacha[1]

[1]Département de Mathématiques, Université de Québec à Montréal (UQÀM), Québec, Canada

[2]Département de Mathématiques et Statistique, Université Laval, Québec, Canada

[3]Département de Médecine Sociale et Préventive, Université Laval, Québec, Canada

[4]Département de Psychatrie et Neuroscience, Université Laval, Québec, Canada

Supplementary Material

QQ-Plots under Null Hypothesis

In this part we present the QQ-Plots under the Null hypothesis from all the different settings. Figures S1 - S2; S3 - S4; S5 - S6 and S7 - S8 show QQ-plots of the p-values of all the considered methods, respectively, where data are generated under the Gaussian copula model, GLMM model, Student copula model and Chi-square copula model.

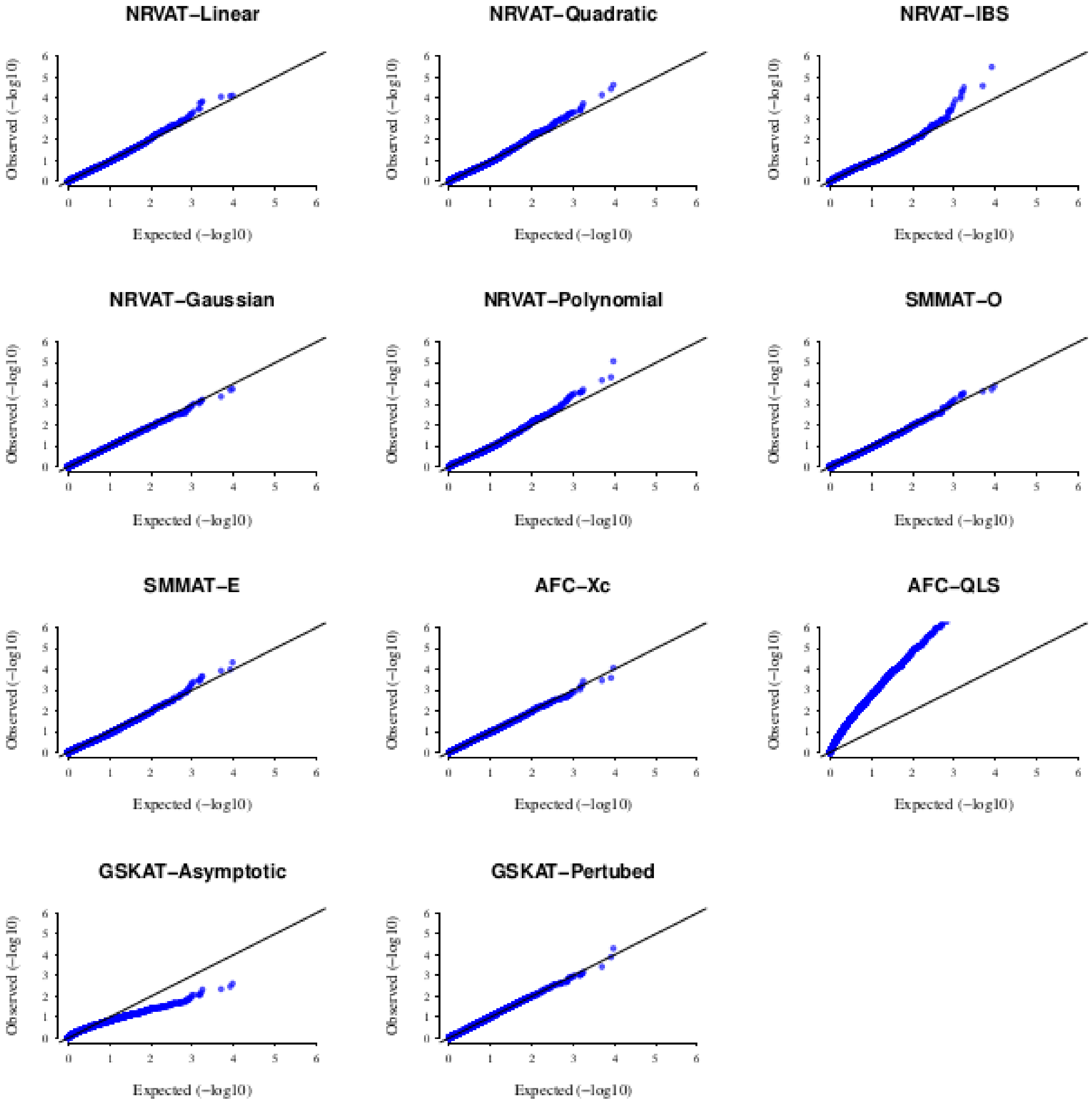

Figure S1: QQ-plot under the null hypothesis of no SNPs/phenotype association ($\tau = 0$), with the heritability parameter $h^2 = 0$, where the data are generated under the Gaussian copula. Results are computed from 10 000 data sets generated under Setting 1. The Compared methods are: NRVAT model with the linear (L), quadratic (Q), identity-by-state (IBS), Gaussian (G), and polynomial (P) kernel matrices; SMMAT model with the hybrid test (O), and the efficient hybrid test (E); AFC model with $\mathcal{X}_c^2$ (Xc), and W_{QLS} (QLS); and gSKAT model with the Asymptotic and Pertubed. SMMAT: variant-set mixed model association tests; AFC: Allele Frequency Comparison tests; gSKAT: burden and kernel-based gene set association tests for binary traits

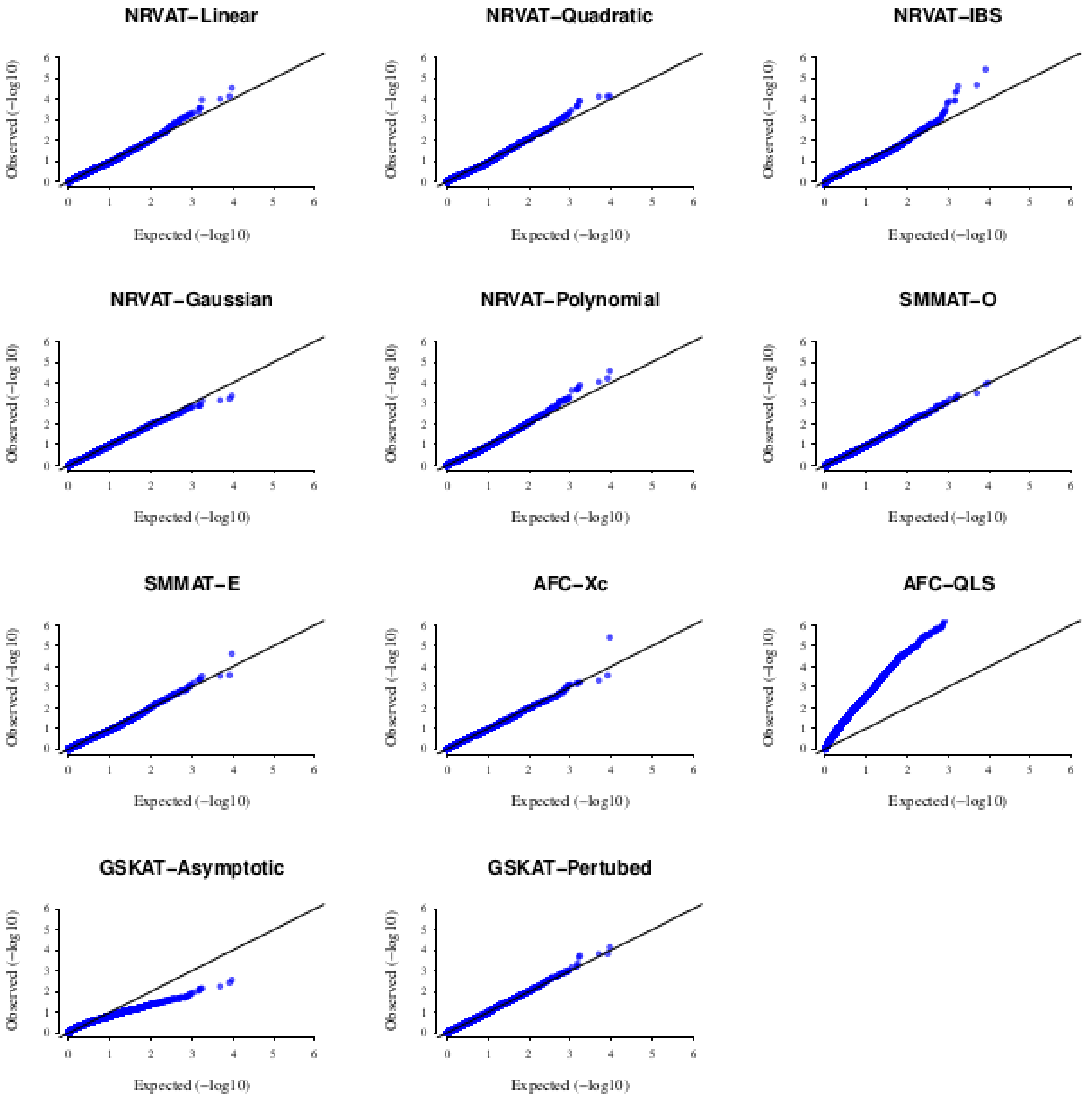

Figure S2: QQ-plot under the null hypothesis of no SNPs/phenotype association ($\tau = 0$), with the heritability parameter $h^2 = 0.2$, where the data are generated under the Gaussian copula. Results are computed from 10 000 data sets generated under Setting 1. The Compared methods are: NRVAT model with the linear (L), quadratic (Q), identity-by-state (IBS), Gaussian (G), and polynomial (P) kernel matrices; SMMAT model with the hybrid test (O), and the efficient hybrid test (E); AFC model with $\mathcal{X}_c^2$ (Xc), and W_{QLS} (QLS); and gSKAT model with the Asymptotic and Pertubed. SMMAT: variant-set mixed model association tests; AFC: Allele Frequency Comparison tests; gSKAT: burden and kernel-based gene set association tests for binary traits

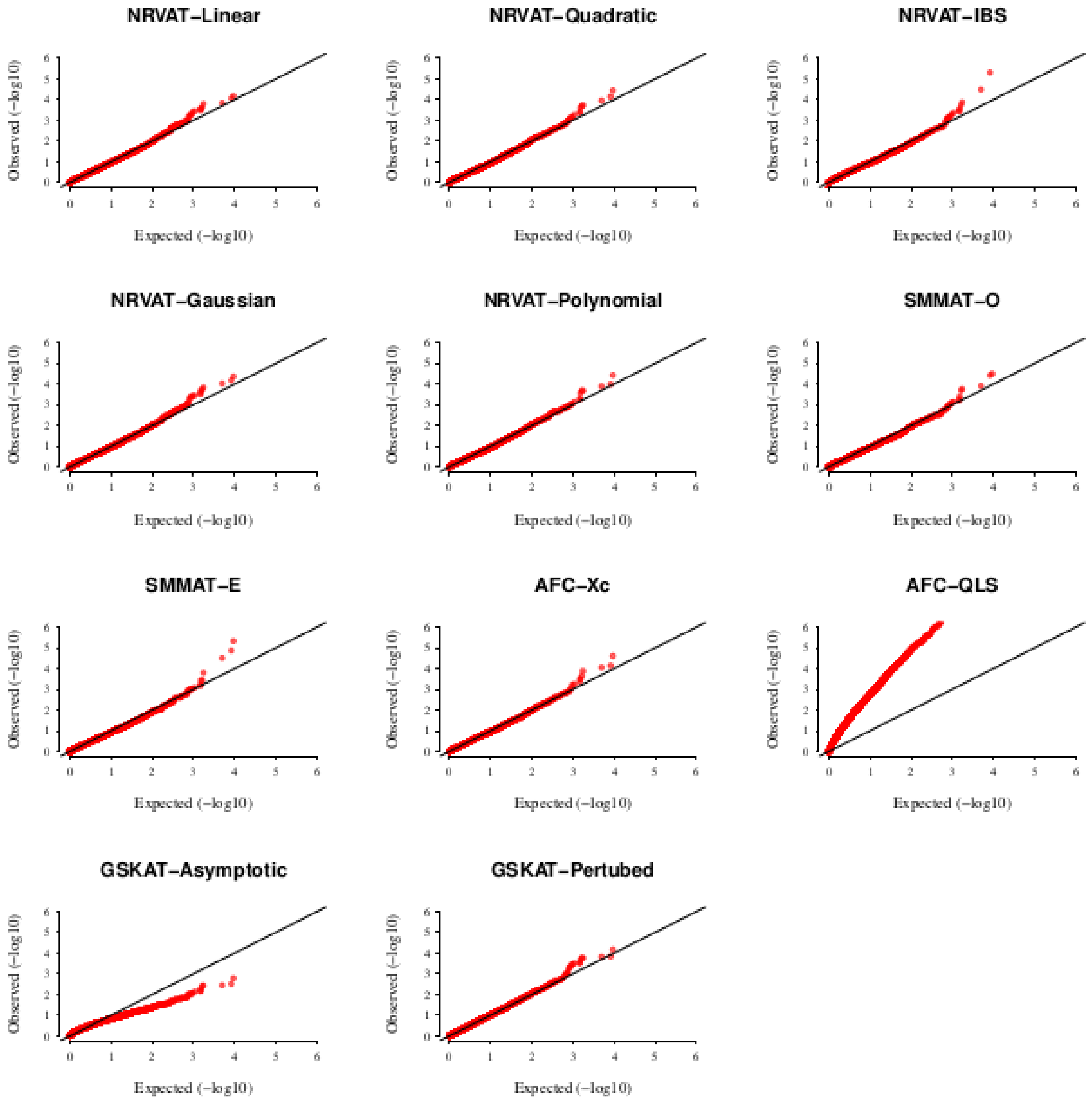

Figure S3: QQ-plot under the null hypothesis of no SNPs/phenotype association ($\tau = 0$), with the heritability parameter $h^2 = 0$, where the data are generated under the generalized linear mixed model (GLMM). Results are computed from 10 000 data sets generated under Setting 2. The Compared methods are: NRVAT model with the linear (L), quadratic (Q), identity-by-state (IBS), Gaussian (G), and polynomial (P) kernel matrices; SMMAT model with the hybrid test (O), and the efficient hybrid test (E); AFC model with $\mathcal{X}_c^2$ (Xc), and W_{QLS} (QLS); and gSKAT model with the Asymptotic and Pertubed. SMMAT: variant-set mixed model association tests; AFC: Allele Frequency Comparison tests; gSKAT: burden and kernel-based gene set association tests for binary traits

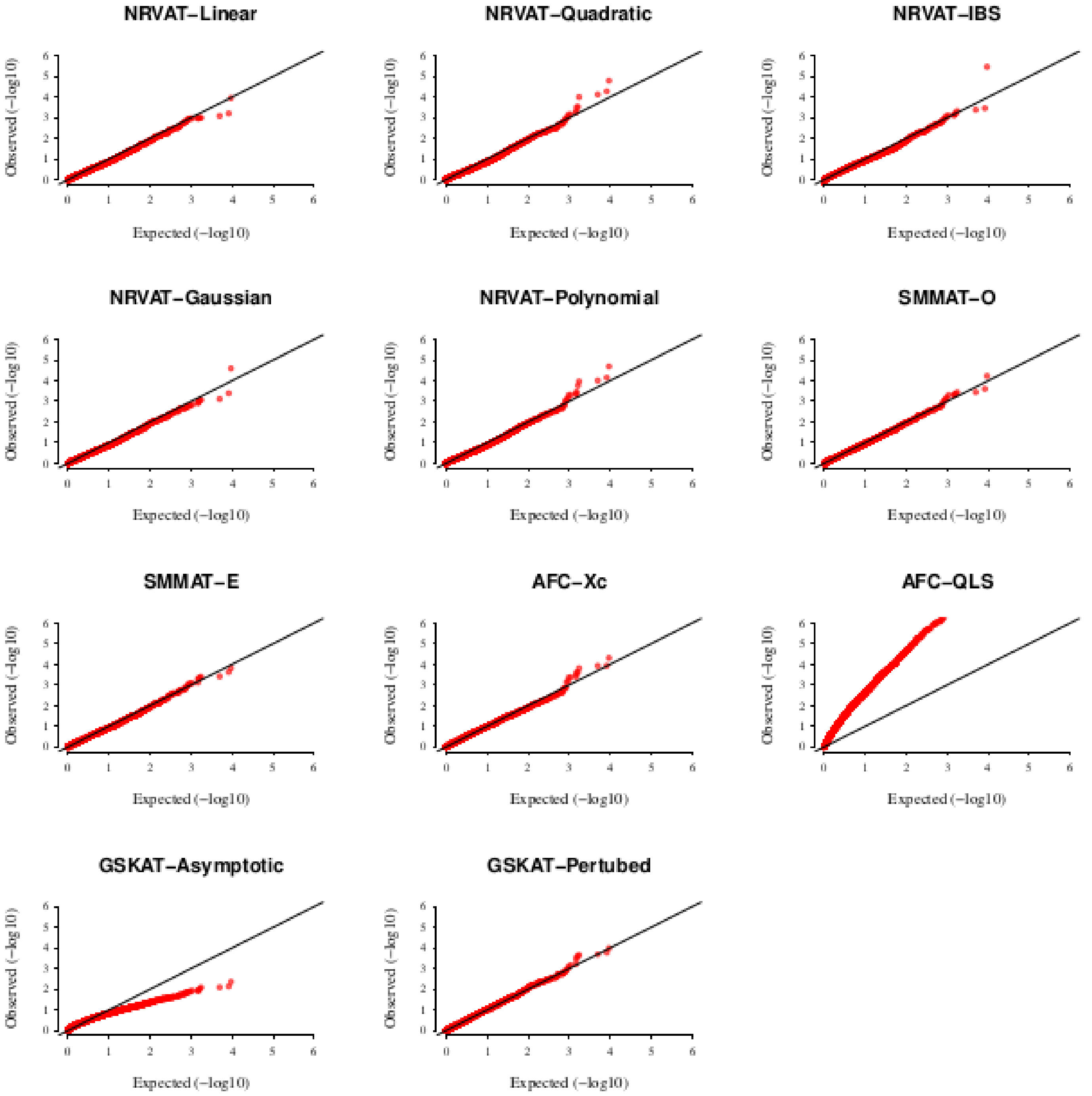

Figure S4: QQ-plot under the null hypothesis of no SNPs/phenotype association ($\tau = 0$), with the heritability parameter $h^2 = 0.2$, where the data are generated under the generalized linear mixed model (GLMM). Results are computed from 10 000 data sets generated under Setting 2. The Compared methods are: NRVAT model with the linear (L), quadratic (Q), identity-by-state (IBS), Gaussian (G), and polynomial (P) kernel matrices; SMMAT model with the hybrid test (O), and the efficient hybrid test (E); AFC model with $\mathcal{X}_c^2$ (Xc), and W_{QLS} (QLS); and gSKAT model with the Asymptotic and Pertubed. SMMAT: variant-set mixed model association tests; AFC: Allele Frequency Comparison tests; gSKAT: burden and kernel-based gene set association tests for binary traits

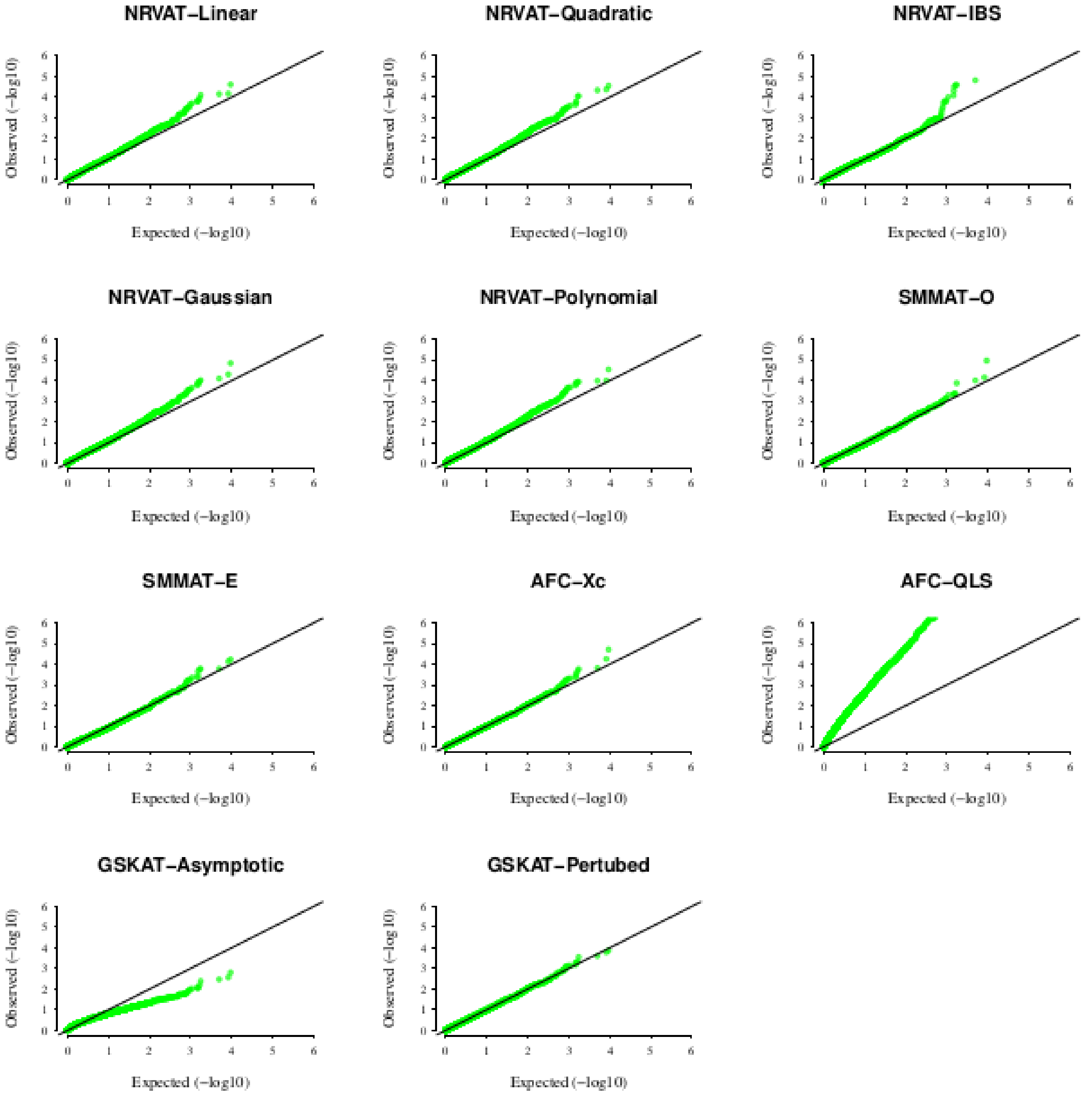

Figure S5: QQ-plot under the null hypothesis of no SNPs/phenotype association ($\tau = 0$), with the heritability parameter $h^2 = 0$, where the data are generated under the Student-t copula model ($df = 3$). Results are computed from 10 000 data sets generated under Scenario 1 of Setting 3. The Compared methods are: NRVAT model with the linear (L), quadratic (Q), identity-by-state (IBS), Gaussian (G), and polynomial (P) kernel matrices; SMMAT model with the hybrid test (O), and the efficient hybrid test (E); AFC model with $\mathcal{X}_c^2$ (Xc), and W_{QLS} (QLS); and gSKAT model with the Asymptotic and Pertubed. SMMAT: variant-set mixed model association tests; AFC: Allele Frequency Comparison tests; gSKAT: burden and kernel-based gene set association tests for binary traits

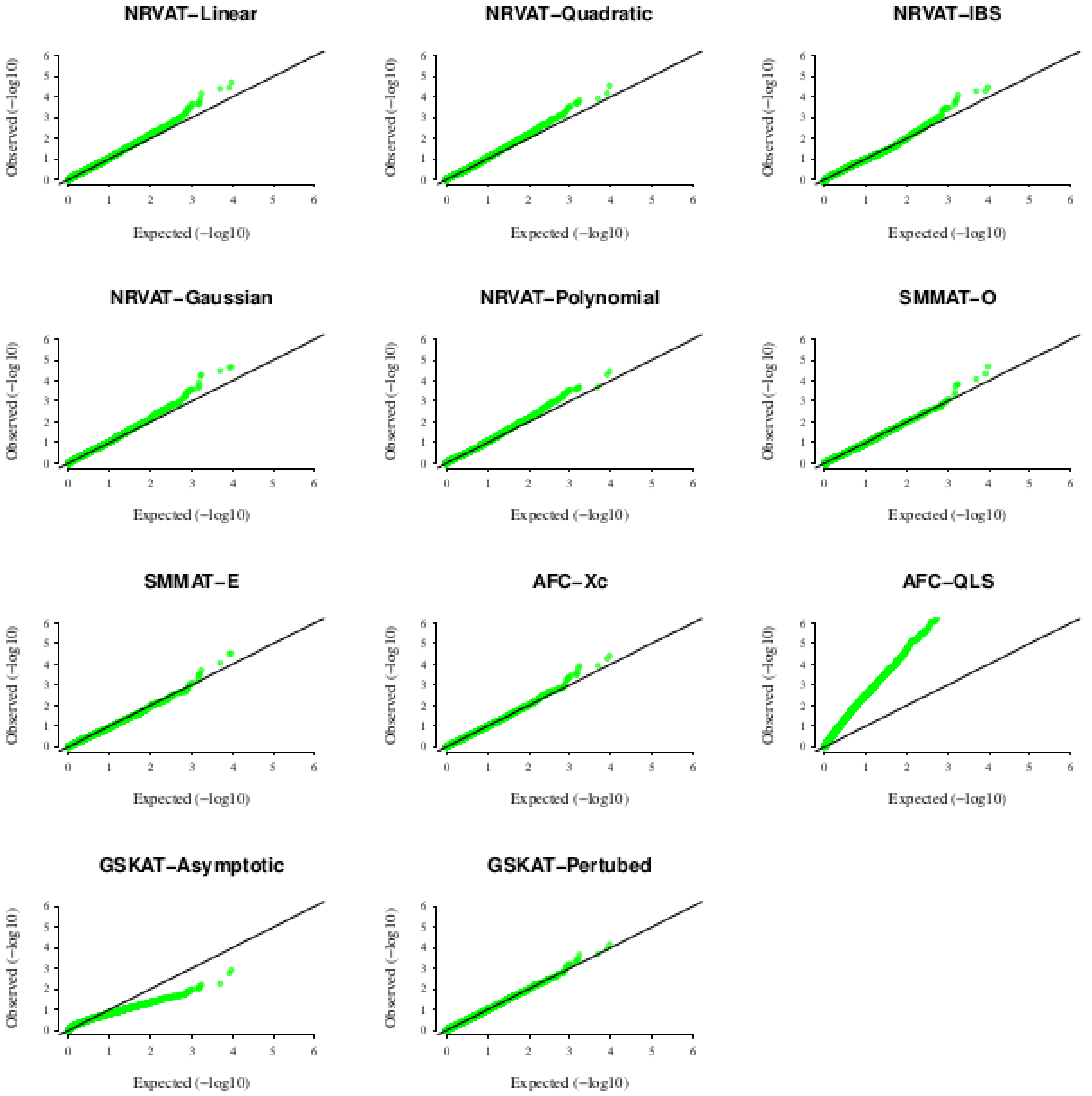

Figure S6: QQ-plot under the null hypothesis of no SNPs/phenotype association ($\tau = 0$), with the heritability parameter $h^2 = 0.2$, where the data are generated under the Student-t copula model ($df = 3$). Results are computed from 10 000 data sets generated under Scenario 1 of Setting 3. The Compared methods are: NRVAT model with the linear (L), quadratic (Q), identity-by-state (IBS), Gaussian (G), and polynomial (P) kernel matrices; SMMAT model with the hybrid test (O), and the efficient hybrid test (E); AFC model with $\mathcal{X}_c^2$ (Xc), and W_{QLS} (QLS); and gSKAT model with the Asymptotic and Pertubed. SMMAT: variant-set mixed model association tests; AFC: Allele Frequency Comparison tests; gSKAT: burden and kernel-based gene set association tests for binary traits

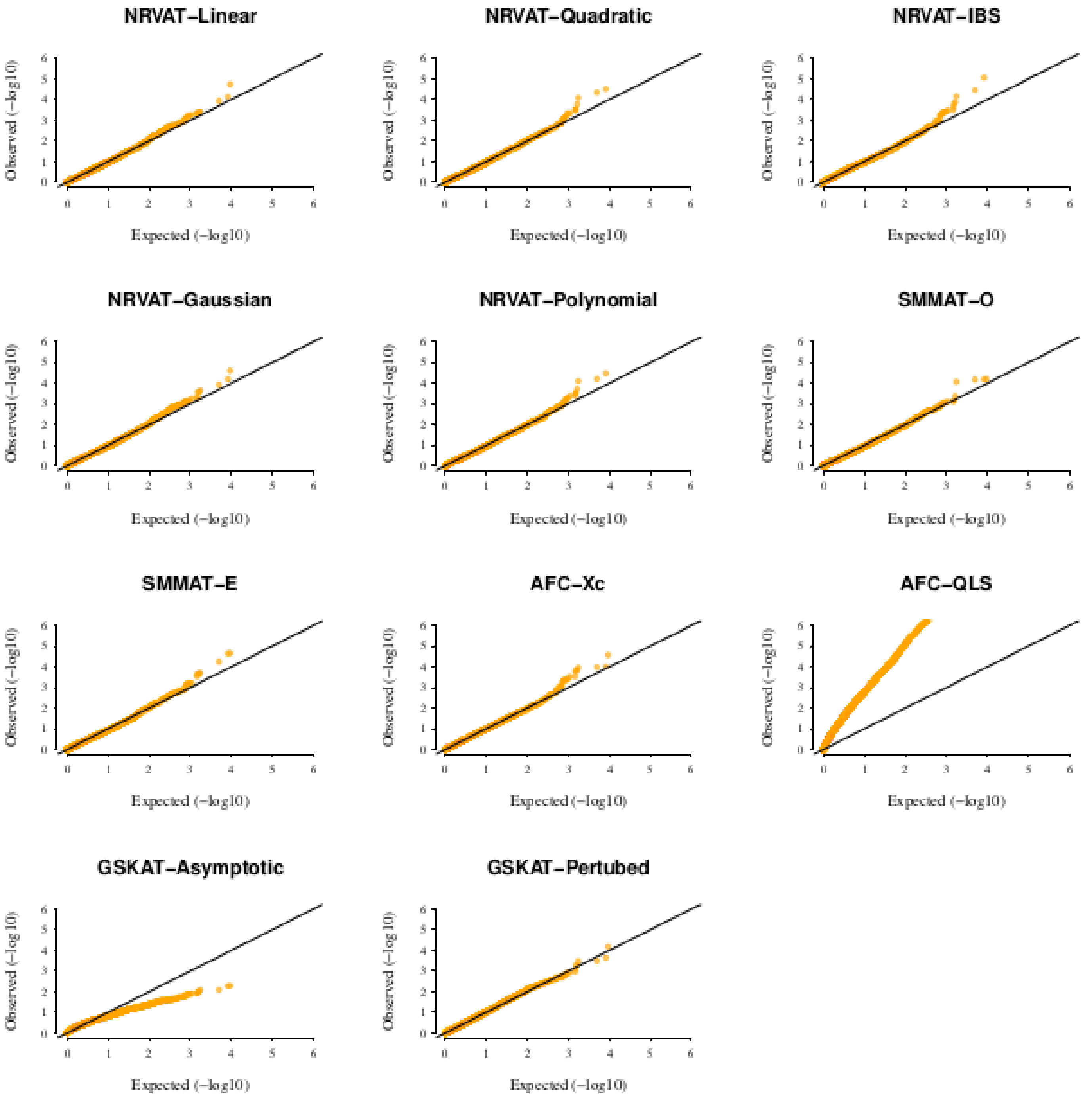

Figure S7: QQ-plot under the null hypothesis of no SNPs/phenotype association ($\tau = 0$), with the heritability parameter $h^2 = 0$, where the data are generated under the Chi-square copula model, with a non-centrality parameter $a = 1$. Results are computed from 10 000 data sets generated under Scenario 2 of Setting 3. The Compared methods are: NRVAT model with the linear (L), quadratic (Q), identity-by-state (IBS), Gaussian (G), and polynomial (P) kernel matrices; SMMAT model with the hybrid test (O), and the efficient hybrid test (E); AFC model with $\mathcal{X}_C^2$ (Xc), and W_{QLS} (QLS); and gSKAT model with the Asymptotic and Pertubed. SMMAT: variant-set mixed model association tests; AFC: Allele Frequency Comparison tests; gSKAT: burden and kernel-based gene set association tests for binary traits

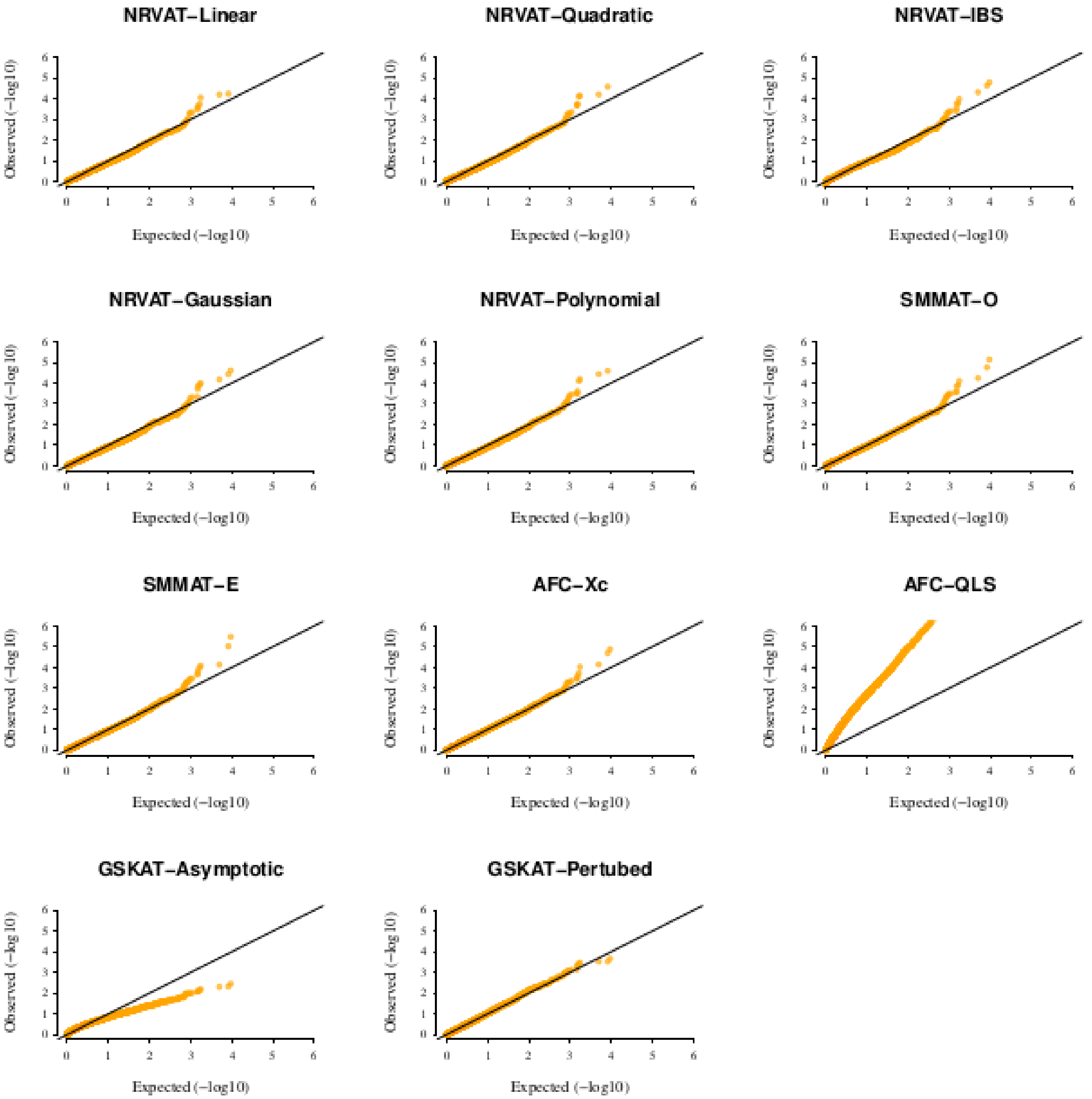

Figure S8: QQ-plot under the null hypothesis of no SNPs/phenotype association ($\tau = 0$), with the heritability parameter $h^2 = 0.2$, where the data are generated under the Chi-square copula model, with a non-centrality parameter $a = 1$. Results are computed from 10 000 data sets generated under Scenario 2 of Setting 3. The Compared methods are: NRVAT model with the linear (L), quadratic (Q), identity-by-state (IBS), Gaussian (G), and polynomial (P) kernel matrices; SMMAT model with the hybrid test (O), and the efficient hybrid test (E); AFC model with $\mathcal{X}_C^2$ (Xc), and W_{QLS} (QLS); and gSKAT model with the Asymptotic and Pertubed. SMMAT: variant-set mixed model association tests; AFC: Allele Frequency Comparison tests; gSKAT: burden and kernel-based gene set association tests for binary traits

Empirical type I error rate under Null Hypothesis

Tables S1 - S4 show Empirical type I error rate of all the considered methods, respectively, where data are generated under the Gaussian copula model, GLMM model, Student copula model and Chi-square copula model.

Table S1: Empirical type I error rate ($\times 100$) under the null hypothesis of no SNPs/phenotype association ($\tau = 0$) where the data are generated under the Gaussian copula model. Results are computed from 10 000 data sets generated under Setting 1. The Compared methods are: NRVAT model with the linear (L), quadratic (Q), identity-by-state (IBS), Gaussian (G), and polynomial (P) kernel matrices; SMMAT model with the hybrid test (O), and the efficient hybrid test (E); AFC model with $\mathcal{X}_C^2$ (Xc), and W_{QLS} (QLS); and gSKAT model with the Asymptotic and Pertubed. SMMAT: variant-set mixed model association tests; AFC: Allele Frequency Comparison tests; gSKAT: burden and kernel-based gene set association tests for binary traits

		NRVAT					SMMAT		AFC		gSKAT	
α	h^2	L	Q	IBS	G	P	O	E	Xc	QLS	Asymptotic	Pertubed
	0	1.18	1.39	0.99	0.91	1.31	1.03	1	1.01	21.13	0.1	1.05
1%	0.2	1.1	1.3	0.99	0.98	1.2	1.11	1.06	0.99	18.29	0.08	1.08
	0.5	1.35	1.51	1.09	0.98	1.44	0.95	0.92	0.96	14.6	0.06	1.19

Table S2: Empirical type I error rate ($\times 100$) under the null hypothesis of no SNPs/phenotype association ($\tau = 0$) where the data are generated under the generalized linear mixed model (GLMM). Results are computed from 10 000 data sets generated under Setting 2. The Compared methods are: NRVAT model with the linear (L), quadratic (Q), identity-by-state (IBS), Gaussian (G), and polynomial (P) kernel matrices; SMMAT model with the hybrid test (O), and the efficient hybrid test (E); AFC model with $\mathcal{X}_C^2$ (Xc), and W_{QLS} (QLS); and gSKAT model with the Asymptotic and Pertubed. SMMAT: variant-set mixed model association tests; AFC: Allele Frequency Comparison tests; gSKAT: burden and kernel-based gene set association tests for binary traits

		NRVAT					SMMAT		AFC		gSKAT	
α	h^2	L	Q	IBS	G	P	O	E	Xc	QLS	Asymptotic	Pertubed
	0	1.04	1.17	0.92	1.11	1.19	0.99	0.84	1.02	21.26	0.15	1.16
1%	0.2	0.82	0.93	0.88	0.92	0.94	0.94	0.92	0.84	20.01	0.05	1.16
	0.5	0.6	0.65	0.65	0.64	0.67	0.93	0.85	0.95	17.55	0.08	1.06

Table S3: Empirical type I error rate (×100) under the null hypothesis of no SNPs/phenotype association ($\tau = 0$) where the data are generated under the Student-t copula ($df = 3$). Results are computed from 10 000 data sets generated under Scenario 1 of Setting 3. The Compared methods are: NRVAT model with the linear (L), quadratic (Q), identity-by-state (IBS), Gaussian (G), and polynomial (P) kernel matrices; SMMAT model with the hybrid test (O), and the efficient hybrid test (E); AFC model with $\mathcal{X}_c^2$ (Xc), and W_{QLS} (QLS); and gSKAT model with the Asymptotic and Pertubed. SMMAT: variant-set mixed model association tests; AFC: Allele Frequency Comparison tests; gSKAT: burden and kernel-based gene set association tests for binary traits

		NRVAT					SMMAT		AFC		gSKAT	
α	h^2	L	Q	IBS	G	P	O	E	Xc	QLS	Asymptotic	Pertubed
	0	1.7	1.75	1.16	1.64	1.75	1.1	1	1.17	18.89	0.09	1.12
1%	0.2	1.5	1.59	1.08	1.41	1.64	1.05	0.99	1.08	16.98	0.09	1.05
	0.5	1.63	1.76	1.05	1.56	1.69	1.09	0.8	1.11	13.44	0.08	1.11

Table S4: Empirical type I error rate (×100) under the null hypothesis of no SNPs/phenotype association ($\tau = 0$) where the data are generated under the Chi-square copula with a non centrality parameter $a = 1$. Results are computed from 10 000 data sets generated under Scenario 2 of Setting 3. The Compared methods are: NRVAT model with the linear (L), quadratic (Q), identity-by-state (IBS), Gaussian (G), and polynomial (P) kernel matrices; SMMAT model with the hybrid test (O), and the efficient hybrid test (E); AFC model with $\mathcal{X}_c^2$ (Xc), and W_{QLS} (QLS); and gSKAT model with the Asymptotic and Pertubed. SMMAT: variant-set mixed model association tests; AFC: Allele Frequency Comparison tests; gSKAT: burden and kernel-based gene set association tests for binary traits

		NRVAT					SMMAT		AFC		gSKAT	
α	h^2	L	Q	IBS	G	P	O	E	Xc	QLS	Asymptotic	Pertubed
	0	1.18	1.13	1.02	1.17	1.06	1.01	1.16	0.97	22.08	0.05	1.23
1%	0.2	0.89	1.01	0.8	0.98	0.93	1.01	1.01	1.06	20.33	0.09	1.31
	0.5	0.7	0.84	0.8	0.77	0.8	1.04	1.11	1.06	17.85	0.14	1.11

Power function

Figure S9 and S10 show the power levels as a function of a grid of values of the variance-component τ for the two values of the polygenic heritability, $h^2 \in \{0, 0.5\}$, under the Gaussian copula model (Setting 1). Again, these figures illustrate the important gain in power achieved by NRVAT with the IBS and the Gaussian Kernel similarity matrices.

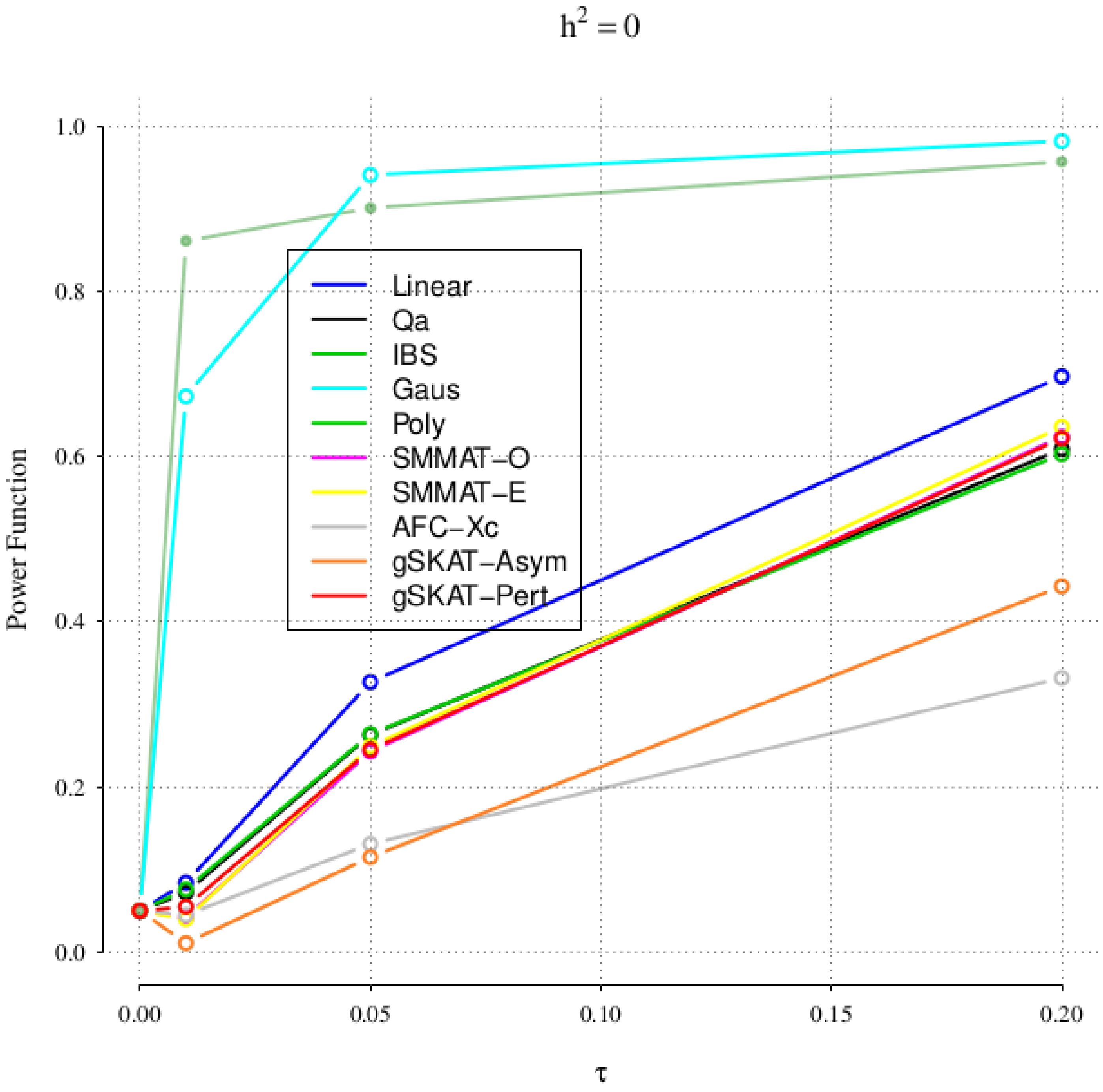

Figure S9: Power function under the alternative hypothesis of SNPs/phenotype association of grid of $\tau \in \{0, 0.01, 0.05, 0.2\}$ for the polygenic heritability parameter $h^2 = 0$ where the data are generated under the Gaussian copula. Results are computed from 1000 data sets generated with twenty-five percent of causal variants taken randomly from the regions size (20) under Setting 1. The Compared methods are: NRVAT model with the linear (L), quadratic (Q), identity-by-state (IBS), Gaussian (G), and polynomial (P) kernel matrices; SMMAT model with the hybrid test (O), and the efficient hybrid test (E); AFC model with $\mathcal{X}_c^2$ (Xc); and gSKAT model with the Asymptotic and Pertubed. SMMAT: variant-set mixed model association tests; AFC: Allele Frequency Comparison tests; gSKAT: burden and kernel-based gene set association tests for binary traits

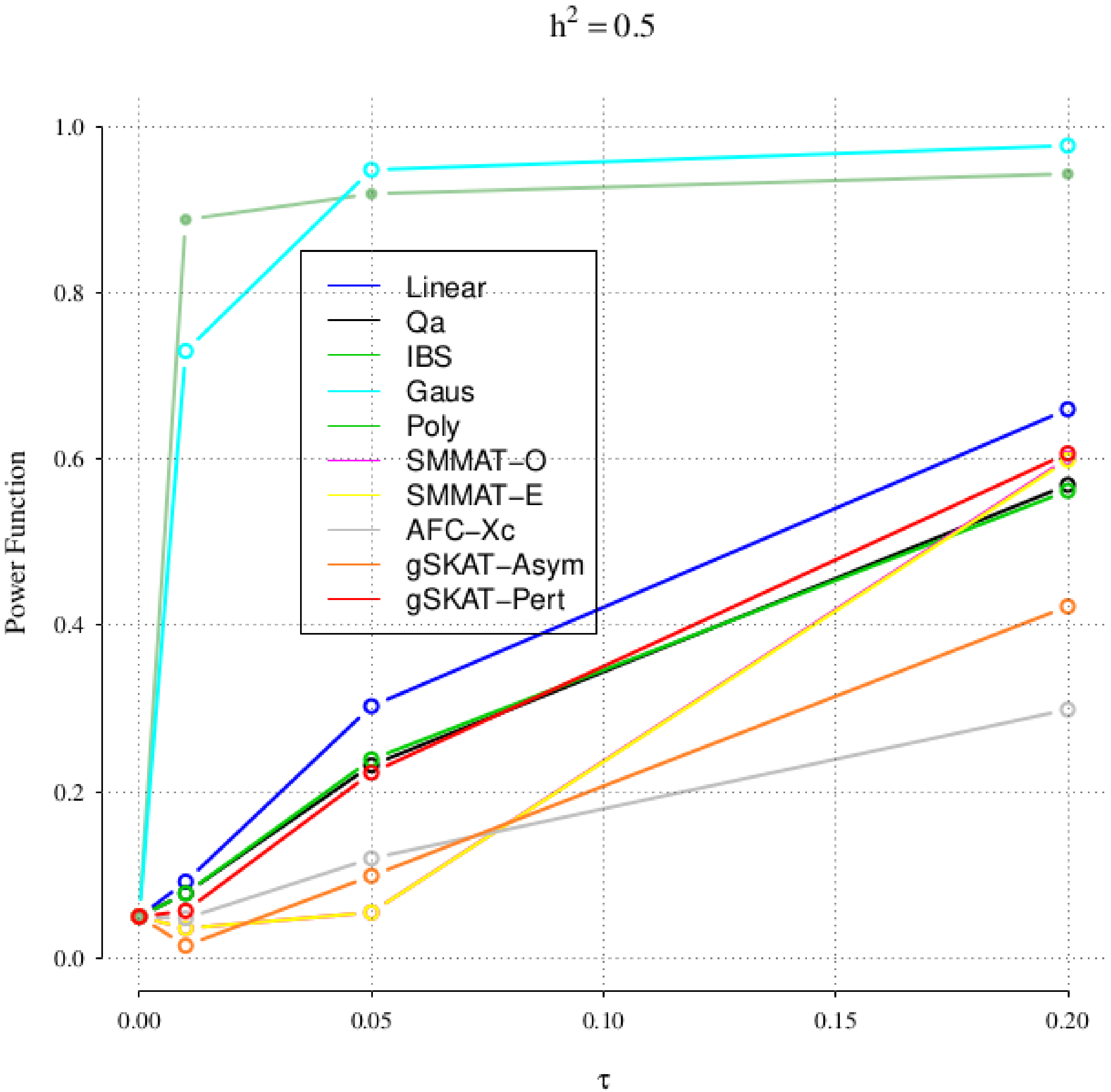

Figure S10: Power function under the alternative hypothesis of SNPs/phenotype association of grid of $\tau \in \{0, 0.01, 0.05, 0.2\}$ for the polygenic heritability parameter $h^2 = 0.5$ where the data are generated under the Gaussian copula. Results are computed from 1000 data sets generated with twenty-five percent of causal variants taken randomly from the regions size (20) under Setting 1. The Compared methods are: NRVAT model with the linear (L), quadratic (Q), identity-by-state (IBS), Gaussian (G), and polynomial (P) kernel matrices; SMMAT model with the hybrid test (O), and the efficient hybrid test (E); AFC model with $\mathcal{X}^2_c$ (Xc); and gSKAT model with the Asymptotic and Pertubed. SMMAT: variant-set mixed model association tests; AFC: Allele Frequency Comparison tests; gSKAT: burden and kernel-based gene set association tests for binary traits

Empirical Bias of the nuissance parameters and the polygenic heritability

Tables S5 - S8 show the empirical bias ($\times 100$) of the nuissance parameters ($\gamma_0, \gamma_1, \gamma_2$) including the intercepte and the polygenic heritability (h^2) under the null hypothesis of no SNP/phenotype association where data are generated under the Gaussian copula model, generalized linear mixed models (GLMM) model, Student copula model and Chi-square copula model, respectively.

Table S5: Empirical bias ($\times 100$) of the nuissance parameters including the intercepte and the polygenic heritability under the null hypothesis of no SNP/phenotype association within gaussian copula where the response variable is computed from 10000 data sets generated under Setting 1, using the polygenic heritability parameter $h^2 \in \{0, 0.2, 0.5\}$; Sd: Standard Deviation; Se: Standard Error.

	γ_0			γ_1			γ_2			$\hat{h}^2$		
h^2	**BIAS**	**Sd**	**Se**	**BIAS**	**Sd**	**Se**	**BIAS**	**Sd**	**Se**	**BIAS**	**Sd**	**Se**
0	-1.47	23.1	23.15	1.12	35.85	35.86	-0.13	22.8	22.8	0.00	0.00	0.04
0.2	-1.68	23.72	23.77	1.36	35.75	35.78	-0.07	22.64	22.64	4.35	4.77	6.46
0.5	-1.83	24.87	24.94	1.53	35.93	35.96	-0.05	22.9	22.9	4.34	4.76	6.44

Table S6: Empirical bias ($\times 100$) of the nuissance parameters including the intercepte and the polygenic heritability under the null hypothesis of no SNP/phenotype association within generalized linear mixed models (GLMM) where the response variable is computed from 10000 data sets generated under Setting 2, using the polygenic heritability parameter $h^2 \in \{0, 0.2, 0.5\}$; Sd: Standard Deviation; Se: Standard Error.

	γ_0			γ_1			γ_2			$\hat{h}^2$		
h^2	**BIAS**	**Sd**	**Se**	**BIAS**	**Sd**	**Se**	**BIAS**	**Sd**	**Se**	**BIAS**	**Sd**	**Se**
0	-0.88	23.05	23.06	0.63	36.02	36.02	0.08	22.77	22.77	0.00	0.00	0.01
0.2	6.81	22.54	23.54	-2.76	35.16	35.27	-3.69	22.5	22.8	2.82	4.32	5.16
0.5	17.06	22.06	27.88	-7.53	34.65	35.45	-8.94	22.46	24.18	1.07	2.93	3.12

Table S7: Empirical bias ($\times 100$) of the nuissance parameters including the intercepte and the polygenic heritability under the Null hypothesis of no SNP/phenotype association within Student-t Copula ($df = 3$) where the response variable is computed from 10000 data sets generated under Setting 3 and scenario 1, using the polygenic heritability parameter $h^2 \in \{0, 0.2, 0.5\}$; Sd: Standard Deviation; Se: Standard Error.

	γ_0			γ_1			γ_2			$\hat{h}^2$		
h^2	**BIAS**	**Sd**	**Se**	**BIAS**	**Sd**	**Se**	**BIAS**	**Sd**	**Se**	**BIAS**	**Sd**	**Se**
0	-1.35	24.49	24.53	1.14	36.05	36.07	-0.06	23.1	23.1	0.01	0.00	0.01
0.2	-1.54	25.15	25.19	1.21	36.07	36.09	0.14	23.14	23.14	5.98	4.71	7.61
0.5	-1.87	26.43	26.5	1.5	36.38	36.41	0.34	23.26	23.26	5.78	4.75	7.48

Table S8: Empirical bias ($\times 100$) of the nuissance parameters including the intercepte and the polygenic heritability under the Null hypothesis of no SNP/phenotype association within Chi-square copula model, with a non-centrality parameter $a = 1$, where the response variable is computed from 10000 data sets generated under Setting 3 and scenario 2, using the polygenic heritability parameter $h^2 \in \{0, 0.2, 0.5\}$; Sd: Standard Deviation; Se: Standard Error.

	γ_0			γ_1			γ_2			$\hat{h}^2$		
h^2	**BIAS**	**Sd**	**Se**	**BIAS**	**Sd**	**Se**	**BIAS**	**Sd**	**Se**	**BIAS**	**Sd**	**Se**
0	-1.14	23.04	23.07	0.56	35.95	35.96	0.33	22.95	22.95	0.00	0.00	0.01
0.2	-0.98	23.24	23.26	0.37	35.93	35.93	0.29	22.84	22.84	3.26	4.5	5.56
0.5	-1.16	23.47	23.49	0.59	35.77	35.77	0.39	22.83	22.84	1.95	3.8	4.27

Additional Simulations

Here, we presented the Algorithm of the four mechanisms used for Additional Simulations.

"Selection" :

• Step 1

Generate the genotypes of at least 2000 families composed of 03 individuals using Simulate3.

• Step 2

i. Simulate the phenotype Y of individuals from each of the 2000 families according to the Gaussian copula.

ii. Retain the first 40 families for which the last individual (the child) has a $Y = 1$.

Use the same procedures ((Step 1 & Step 2)) to obtain the 40 families composed of 04 individuals and those composed of 08 individuals.

iii. Use the new genotypes and Y of the three categories of 40 families to determine the results.

"MAR (Missing At Random)" : here, the deletion of the parental lines (to obtain the missing data at the level of the families in which we have 04 and 08 members respectively) depends on the phenotype Y of the child, i.e., the last member of the family.

• Step 1

Generate according to Simulate3 the genotypes for a total number of 120 families of which 40 are composed of 03 individuals, 40 others are composed of 04 individuals and the remaining 40 are composed of 08 individuals.

• Step 2

i. Simulate the Y according to the Gaussian copula

ii. For families composed of 04 people, delete the first two rows (parents) of the family if the last member has a $Y = 0$. Otherwise ($Y = 1$), keep the whole family.

iii. For families made up of 08 members, delete the first two lines (grandparents) of the family if the last member has a $Y = 0$. Otherwise, keep the whole family.

iv. Use the new genotypes and Y (obtained after removing these parental lines) to determine the results.

"MCAR (Missing Complete At Random)" : we assume that 20% of the data is missing at the level of the families in which we have 04 and 08 members respectively.

• Step 1

Generate according to Simulate3 the genotypes for a total number of 120 families of which 40 are composed of 03 individuals, 40 others are composed of 04 individuals and the remaining 40 are composed of 08 individuals.

• Step 2

i. Simulate the phenotype Y according to the Gaussian copula

ii. Randomly delete a few parental lines (this for families of 04 and 08 people) with a probability of success equal to 0.2 (20%)

iii. Use the new genotypes and Y (obtained after removing these parental lines) to determine the results.

QQ-Plots under H_0 of selection bias and missing genotypes

Figures S11 - S13; S14 - S16 and S17 - S19 show QQ-plots of the p-values of NRVAT model with the linear (L), quadratic (Q), identity-by-state (IBS), Gaussian (G), and polynomial (P) kernel matrices under the selection bias; the missing at random (MAR) approaches and the missing completely at random (MCAR), respectively, where data are generated under the Gaussian copula model.

All_Selction_Bias_hs_0.png

Figure S11: selection bias: QQ-plot under the null hypothesis of no SNPs/phenotype association ($\tau = 0$), with the heritability parameter $h^2 = 0$, where the data are generated under the Gaussian copula model. Results are computed from 10 000 data sets generated under the selection bias. The Compared methods are: NRVAT model with the linear (L), quadratic (Q), identity-by-state (IBS), Gaussian (G), and polynomial (P) kernel matrices; SMMAT model with the hybrid test (O), and the efficient hybrid test (E); AFC model with $\mathcal{X}_c^2$ (Xc), and W_{QLS} (QLS); and gSKAT model with the Asymptotic and Pertubed. SMMAT: variant-set mixed model association tests; AFC: Allele Frequency Comparison tests; gSKAT: burden and kernel-based gene set association tests for binary traits

All_Selction_Bias_hs_02.png

Figure S12: selection bias: QQ-plot under the null hypothesis of no SNPs/phenotype association ($\tau = 0$), with the heritability parameter $h^2 = 0.2$, where the data are generated under the Gaussian copula model. Results are computed from 10 000 data sets generated under the selection bias. The Compared methods are: NRVAT model with the linear (L), quadratic (Q), identity-by-state (IBS), Gaussian (G), and polynomial (P) kernel matrices; SMMAT model with the hybrid test (O), and the efficient hybrid test (E); AFC model with $\mathcal{X}_c^2$ (Xc), and W_{QLS} (QLS); and gSKAT model with the Asymptotic and Pertubed. SMMAT: variant-set mixed model association tests; AFC: Allele Frequency Comparison tests; gSKAT: burden and kernel-based gene set association tests for binary traits

All_Selction_Bias_hs_05.png

Figure S13: selection bias: QQ-plot under the null hypothesis of no SNPs/phenotype association ($\tau = 0$), with the heritability parameter $h^2 = 0.5$, where the data are generated under the Gaussian copula model. Results are computed from 10 000 data sets generated under the selection bias. The Compared methods are: NRVAT model with the linear (L), quadratic (Q), identity-by-state (IBS), Gaussian (G), and polynomial (P) kernel matrices; SMMAT model with the hybrid test (O), and the efficient hybrid test (E); AFC model with $\mathcal{X}_c^2$ (Xc), and W_{QLS} (QLS); and gSKAT model with the Asymptotic and Pertubed. SMMAT: variant-set mixed model association tests; AFC: Allele Frequency Comparison tests; gSKAT: burden and kernel-based gene set association tests for binary traits

All_MAR_hs_0.png

Figure S14: MAR: QQ-plot under the null hypothesis of no SNPs/phenotype association ($\tau = 0$), with the heritability parameter $h^2 = 0$, where the data are generated under the Gaussian copula model. Results are computed from 10 000 data sets generated under the missing at random. The Compared methods are: NRVAT model with the linear (L), quadratic (Q), identity-by-state (IBS), Gaussian (G), and polynomial (P) kernel matrices; SMMAT model with the hybrid test (O), and the efficient hybrid test (E); AFC model with $\mathcal{X}_c^2$ (Xc), and W_{QLS} (QLS); and gSKAT model with the Asymptotic and Pertubed. SMMAT: variant-set mixed model association tests; AFC: Allele Frequency Comparison tests; gSKAT: burden and kernel-based gene set association tests for binary traits

All_MAR_hs_02.png

Figure S15: MAR: QQ-plot under the null hypothesis of no SNPs/phenotype association ($\tau = 0$), with the heritability parameter $h^2 = 0.2$, where the data are generated under the Gaussian copula model. Results are computed from 10 000 data sets generated under the missing at random. The Compared methods are: NRVAT model with the linear (L), quadratic (Q), identity-by-state (IBS), Gaussian (G), and polynomial (P) kernel matrices; SMMAT model with the hybrid test (O), and the efficient hybrid test (E); AFC model with $\mathcal{X}_c^2$ (Xc), and W_{QLS} (QLS); and gSKAT model with the Asymptotic and Pertubed. SMMAT: variant-set mixed model association tests; AFC: Allele Frequency Comparison tests; gSKAT: burden and kernel-based gene set association tests for binary traits

All_MAR_hs_05.png

Figure S16: MAR: QQ-plot under the null hypothesis of no SNPs/phenotype association ($\tau = 0$), with the heritability parameter $h^2 = 0.5$, where the data are generated under the Gaussian copula model. Results are computed from 10 000 data sets generated under the missing at random. The Compared methods are: NRVAT model with the linear (L), quadratic (Q), identity-by-state (IBS), Gaussian (G), and polynomial (P) kernel matrices; SMMAT model with the hybrid test (O), and the efficient hybrid test (E); AFC model with $\mathcal{X}_c^2$ (Xc), and W_{QLS} (QLS); and gSKAT model with the Asymptotic and Pertubed. SMMAT: variant-set mixed model association tests; AFC: Allele Frequency Comparison tests; gSKAT: burden and kernel-based gene set association tests for binary traits

All_MCAR_hs_0.png

Figure S17: MCAR: QQ-plot under the null hypothesis of no SNPs/phenotype association ($\tau = 0$), with the heritability parameter $h^2 = 0$, where the data are generated under the Gaussian copula model. Results are computed from 10 000 data sets generated under the missing completely at random. The Compared methods are: NRVAT model with the linear (L), quadratic (Q), identity-by-state (IBS), Gaussian (G), and polynomial (P) kernel matrices; SMMAT model with the hybrid test (O), and the efficient hybrid test (E); AFC model with $\mathcal{X}_c^2$ (Xc), and W_{QLS} (QLS); and gSKAT model with the Asymptotic and Pertubed. SMMAT: variant-set mixed model association tests; AFC: Allele Frequency Comparison tests; gSKAT: burden and kernel-based gene set association tests for binary traits

All_MCAR_hs_02.png

Figure S18: MCAR: QQ-plot under the null hypothesis of no SNPs/phenotype association ($\tau = 0$), with the heritability parameter $h^2 = 0.2$, where the data are generated under the Gaussian copula model. Results are computed from 10 000 data sets generated under the missing completely at random. The Compared methods are: NRVAT model with the linear (L), quadratic (Q), identity-by-state (IBS), Gaussian (G), and polynomial (P) kernel matrices; SMMAT model with the hybrid test (O), and the efficient hybrid test (E); AFC model with $\mathcal{X}_c^2$ (Xc), and W_{QLS} (QLS); and gSKAT model with the Asymptotic and Pertubed. SMMAT: variant-set mixed model association tests; AFC: Allele Frequency Comparison tests; gSKAT: burden and kernel-based gene set association tests for binary traits

All_MCAR_hs_05.png

Figure S19: MCAR: QQ-plot under the null hypothesis of no SNPs/phenotype association ($\tau = 0$), with the heritability parameter $h^2 = 0.5$, where the data are generated under the Gaussian copula model. Results are computed from 10 000 data sets generated under the missing completely at random for. The Compared methods are: NRVAT model with the linear (L), quadratic (Q), identity-by-state (IBS), Gaussian (G), and polynomial (P) kernel matrices; SMMAT model with the hybrid test (O), and the efficient hybrid test (E); AFC model with $\mathcal{X}_c^2$ (Xc), and W_{QLS} (QLS); and gSKAT model with the Asymptotic and Pertubed. SMMAT: variant-set mixed model association tests; AFC: Allele Frequency Comparison tests; gSKAT: burden and kernel-based gene set association tests for binary traits

Empirical Bias of the nuissance parameters and the polygenic heritability under Additional Simulations

Tables S9 - S10 show the empirical bias ($\times 100$) of the nuissance parameters ($\gamma_0, \gamma_1, \gamma_2$) including the intercepte and the polygenic heritability (h^2) for the null hypothesis of no SNP/phenotype association under the selection bias; the missing at random (MAR) and the missing completely at random (MCAR) approaches, respectively, where data are generated with the Gaussian copula model.

Table S9: selection bias: Empirical bias ($\times 100$) of the nuissance parameters including the intercepte and the polygenic heritability under the null hypothesis of no SNP/phenotype association within Gaussian copula model where the response variable is computed from 10000 data sets generated where the data are generated under the selection bias using the polygenic heritability parameter $h^2 \in \{0, 0.2, 0.5\}$; Sd: Standard Deviation; Se: Standard Error.

	γ_0			γ_1			γ_2			h^2		
h^2	**BIAS**	**Sd**	**Se**	**BIAS**	**Sd**	**Se**	**BIAS**	**Sd**	**Se**	**BIAS**	**Sd**	**Se**
0	73.93	18.55	76.22	0.85	30.22	30.23	0.45	20.34	20.34	0.00	0.00	0.00
0.2	86.7	18.5	88.66	1.76	30.07	30.12	2.00	20.66	20.76	0.00	0.00	0.00
0.5	106.03	18.91	107.7	3.67	29.53	29.75	4.49	20.8	21.28	0.00	0.00	0.00

Table S10: MAR: Empirical bias ($\times 100$) of the nuissance parameters including the intercepte and the polygenic heritability under the Null hypothesis of no SNP/phenotype association within Gaussian copula model where the response variable is computed from 10000 data sets generated under the missing at random (MAR), using the polygenic heritability parameter $h^2 \in \{0, 0.2, 0.5\}$; Sd: Standard Deviation; Se: Standard Error.

	γ_0			γ_1			γ_2			h^2		
h^2	**BIAS**	**Sd**	**Se**	**BIAS**	**Sd**	**Se**	**BIAS**	**Sd**	**Se**	**BIAS**	**Sd**	**Se**
0	0.14	23.42	23.42	-0.79	36.18	36.19	-1.15	22.92	22.95	0.00	0.01	0.00
0.2	-0.62	24.16	24.17	-0.79	36.39	36.4	-1.12	22.93	22.95	4.28	4.76	6.41
0.5	-1.78	25.27	25.33	-1.1	36.66	36.68	-0.97	23.06	23.08	4.24	4.75	6.37

Table S11: MCAR: Empirical bias (×100) of the nuissance parameters including the intercepte and the polygenic heritability under the null hypothesis of no SNP/phenotype association within Gaussian copula model where the response variable is computed from 10000 data sets generated under the missing completely at random (MCAR), using the polygenic heritability parameter $h^2 \in \{0, 0.2, 0.5\}$; Sd: Standard Deviation; Se: Standard Error.

	γ_0			γ_1			γ_2			h^2		
h^2	**BIAS**	**Sd**	**Se**	**BIAS**	**Sd**	**Se**	**BIAS**	**Sd**	**Se**	**BIAS**	**Sd**	**Se**
0	-1.5	25.68	25.72	0.95	39.66	39.67	0.06	25.44	25.44	0.00	0.01	6.42
0.2	-1.64	26.36	26.41	0.94	39.77	39.78	0.14	25.4	25.4	4.28	4.76	0.00
0.5	-1.85	27.48	27.54	1.1	40.1	40.12	0.21	25.53	25.53	4.33	4.76	6.46

Sensitivity analysis (*Setting 1*): QQ-Plots under null hypothesis with larger d^2.

Figures S20 - S22 and S23 - S25 show QQ-plots of all the considered methods, for $d^2 = 0.25$, and $d^2 = 0.36$, respectively, where data are generated under the Gaussian copula model.

Figure S20: QQ-plot under the null hypothesis of no SNPs/phenotype association ($\tau = 0$), with the heritability parameter $h^2 = 0$, where the data are generated under the Gaussian copula model for $d^2 = 0.25$. Results are computed from 10 000 data sets. The Compared methods are: NRVAT model with the linear (L), quadratic (Q), identity-by-state (IBS), Gaussian (G), and polynomial (P) kernel matrices; SMMAT model with the hybrid test (O), and the efficient hybrid test (E); AFC model with $\mathcal{X}_c^2$ (Xc), and W_{QLS} (QLS); and gSKAT model with the Asymptotic and Pertubed. SMMAT: variant-set mixed model association tests; AFC: Allele Frequency Comparison tests; gSKAT: burden and kernel-based gene set association tests for binary traits

Figure S21: QQ-plot under the null hypothesis of no SNPs/phenotype association ($\tau = 0$), with the heritability parameter $h^2 = 0.2$, where the data are generated under the Gaussian copula model for $d^2 = 0.25$. Results are computed from 10 000 data sets. The Compared methods are: NRVAT model with the linear (L), quadratic (Q), identity-by-state (IBS), Gaussian (G), and polynomial (P) kernel matrices; SMMAT model with the hybrid test (O), and the efficient hybrid test (E); AFC model with $\mathcal{X}_c^2$ (Xc), and W_{QLS} (QLS); and gSKAT model with the Asymptotic and Pertubed. SMMAT: variant-set mixed model association tests; AFC: Allele Frequency Comparison tests; gSKAT: burden and kernel-based gene set association tests for binary traits

Figure S22: QQ-plot under the null hypothesis of no SNPs/phenotype association ($\tau = 0$), with the heritability parameter $h^2 = 0.5$, where the data are generated under the Gaussian copula model for $d^2 = 0.25$. Results are computed from 10 000 data sets. The Compared methods are: NRVAT model with the linear (L), quadratic (Q), identity-by-state (IBS), Gaussian (G), and polynomial (P) kernel matrices; SMMAT model with the hybrid test (O), and the efficient hybrid test (E); AFC model with $\mathcal{X}_c^2$ (Xc), and W_{QLS} (QLS); and gSKAT model with the Asymptotic and Pertubed. SMMAT: variant-set mixed model association tests; AFC: Allele Frequency Comparison tests; gSKAT: burden and kernel-based gene set association tests for binary traits

Figure S23: QQ-plot under the null hypothesis of no SNPs/phenotype association ($\tau = 0$), with the heritability parameter $h^2 = 0$, where the data are generated under the Gaussian copula model for $d^2 = 0.36$. Results are computed from 10 000 data sets. The Compared methods are: NRVAT model with the linear (L), quadratic (Q), identity-by-state (IBS), Gaussian (G), and polynomial (P) kernel matrices; SMMAT model with the hybrid test (O), and the efficient hybrid test (E); AFC model with $\mathcal{X}_c^2$ (Xc), and W_{QLS} (QLS); and gSKAT model with the Asymptotic and Pertubed. SMMAT: variant-set mixed model association tests; AFC: Allele Frequency Comparison tests; gSKAT: burden and kernel-based gene set association tests for binary traits

Figure S24: QQ-plot under the null hypothesis of no SNPs/phenotype association ($\tau = 0$), with the heritability parameter $h^2 = 0.2$, where the data are generated under the Gaussian copula model for $d^2 = 0.36$. Results are computed from 10 000 data sets. The Compared methods are: NRVAT model with the linear (L), quadratic (Q), identity-by-state (IBS), Gaussian (G), and polynomial (P) kernel matrices; SMMAT model with the hybrid test (O), and the efficient hybrid test (E); AFC model with $\mathcal{X}_c^2$ (Xc), and W_{QLS} (QLS); and gSKAT model with the Asymptotic and Pertubed. SMMAT: variant-set mixed model association tests; AFC: Allele Frequency Comparison tests; gSKAT: burden and kernel-based gene set association tests for binary traits

Figure S25: QQ-plot under the null hypothesis of no SNPs/phenotype association ($\tau = 0$), with the heritability parameter $h^2 = 0.5$, where the data are generated under the Gaussian copula model for $d^2 = 0.36$. Results are computed from 10 000 data sets. The Compared methods are: NRVAT model with the linear (L), quadratic (Q), identity-by-state (IBS), Gaussian (G), and polynomial (P) kernel matrices; SMMAT model with the hybrid test (O), and the efficient hybrid test (E); AFC model with $\mathcal{X}_c^2$ (Xc), and W_{QLS} (QLS); and gSKAT model with the Asymptotic and Pertubed. SMMAT: variant-set mixed model association tests; AFC: Allele Frequency Comparison tests; gSKAT: burden and kernel-based gene set association tests for binary traits

Sensitivity analysis (*Setting 1*): Empirical type I error rate under null hypothesis with larger d^2.

Tables S12 - S13 show Empirical type I error rate of all the considered methods, for $d^2 = 0.25$, and $d^2 = 0.36$, respectively, where data are generated under the Gaussian copula model.

Table S12: Empirical type I error rate ($\times 100$) under the null hypothesis of no SNPs/phenotype association ($\tau = 0$) where the data are generated under the Gaussian copula model for $d^2 = 0.25$. Results are computed from 10 000 data sets generated under Setting 1. The Compared methods are: NRVAT model with the linear (L), quadratic (Q), identity-by-state (IBS), Gaussian (G), and polynomial (P) kernel matrices; SMMAT model with the hybrid test (O), and the efficient hybrid test (E); AFC model with $\mathcal{X}_c^2$ (Xc), and W_{QLS} (QLS); and gSKAT model with the Asymptotic and Pertubed. SMMAT: variant-set mixed model association tests; AFC: Allele Frequency Comparison tests; gSKAT: burden and kernel-based gene set association tests for binary traits

		NRVAT					SMMAT		AFC		gSKAT	
α	h^2	L	Q	IBS	G	P	O	E	Xc	QLS	Asymptotic	Pertubed
1%	0	1.21	1.35	0.99	0.92	1.35	0.84	0.94	0.86	18.79	0.11	1.29
	0.2	1.18	1.33	0.99	0.93	1.29	1.00	1.08	0.93	15.55	0.09	1.29
	0.5	1.35	1.51	1.09	0.98	1.44	0.95	0.92	0.96	14.6	0.06	1.19

Table S13: Empirical type I error rate ($\times 100$) under the null hypothesis of no SNPs/phenotype association ($\tau = 0$) where the data are generated under the Gaussian copula model for $d^2 = 0.36$. Results are computed from 10 000 data sets generated under Setting 1. The Compared methods are: NRVAT model with the linear (L), quadratic (Q), identity-by-state (IBS), Gaussian (G), and polynomial (P) kernel matrices; SMMAT model with the hybrid test (O), and the efficient hybrid test (E); AFC model with $\mathcal{X}_c^2$ (Xc), and W_{QLS} (QLS); and gSKAT model with the Asymptotic and Pertubed. SMMAT: variant-set mixed model association tests; AFC: Allele Frequency Comparison tests; gSKAT: burden and kernel-based gene set association tests for binary traits

		NRVAT					SMMAT		AFC		gSKAT	
α	h^2	L	Q	IBS	G	P	O	E	Xc	QLS	Asymptotic	Pertubed
1%	0	1.26	1.46	0.99	1.07	1.39	0.89	1.03	0.99	16.37	0.09	1.35
	0.2	1.19	1.46	0.99	1.00	1.39	0.99	1.00	0.95	14.32	0.07	1.36
	0.5	1.46	1.58	1.09	1.00	1.58	1.13	0.98	1.15	11.66	0.07	1.26
